# Phylogenetic assembly of methanogenesis regulates methane yield in food-waste anaerobic digestion

**DOI:** 10.1093/ismejo/wrag083

**Published:** 2026-04-11

**Authors:** Bo Zhao, Xingsheng Yang, Kai Feng, Jie Wang, Mingqian Liu, Yingcheng Wang, Danrui Wang, Xi Peng, Qing He, Yanjuan Lu, Hassan Waseem, Shang Wang, Ye Deng

**Affiliations:** State Key Laboratory of Regional Environment and Sustainability, Research Center for Eco-Environmental Sciences, Chinese Academy of Sciences, Beijing 100085, China; University of Chinese Academy of Sciences, Beijing 100049, China; State Key Laboratory of Regional Environment and Sustainability, Research Center for Eco-Environmental Sciences, Chinese Academy of Sciences, Beijing 100085, China; University of Chinese Academy of Sciences, Beijing 100049, China; State Key Laboratory of Regional Environment and Sustainability, Research Center for Eco-Environmental Sciences, Chinese Academy of Sciences, Beijing 100085, China; University of Chinese Academy of Sciences, Beijing 100049, China; State Key Laboratory of Biogeology and Environmental Geology, China University of Geosciences, Beijing 100053, China; State Key Laboratory of Regional Environment and Sustainability, Research Center for Eco-Environmental Sciences, Chinese Academy of Sciences, Beijing 100085, China; University of Chinese Academy of Sciences, Beijing 100049, China; Qinghai Provincial Key Laboratory of Restoration Ecology in Cold Regions, Northwest Institute of Plateau Biology, Chinese Academy of Sciences, Xining 810008, China; Soil Ecology Lab, Jiangsu Collaborative Innovation Center for Solid Organic Waste Resource Utilization and Jiangsu Key Laboratory for Solid Organic Waste Utilization, Nanjing Agriculture University, Nanjing 210095, China; State Key Laboratory of Regional Environment and Sustainability, Research Center for Eco-Environmental Sciences, Chinese Academy of Sciences, Beijing 100085, China; University of Chinese Academy of Sciences, Beijing 100049, China; State Key Laboratory of Regional Environment and Sustainability, Research Center for Eco-Environmental Sciences, Chinese Academy of Sciences, Beijing 100085, China; University of Chinese Academy of Sciences, Beijing 100049, China; Fairyland Environmental Technology Co., Ltd, Beijing 100085, China; Department of Civil and Environmental Engineering, Carleton University, 1125 Colonel By Dr, Ottawa, Ontario K1S 5B6, Canada; State Key Laboratory of Regional Environment and Sustainability, Research Center for Eco-Environmental Sciences, Chinese Academy of Sciences, Beijing 100085, China; University of Chinese Academy of Sciences, Beijing 100049, China; State Key Laboratory of Regional Environment and Sustainability, Research Center for Eco-Environmental Sciences, Chinese Academy of Sciences, Beijing 100085, China; University of Chinese Academy of Sciences, Beijing 100049, China

**Keywords:** anaerobic digestion, methane production, metagenomics, methanogenic genes, trait phylogeny, functional robustness

## Abstract

Anaerobic digestion (AD) of food waste (FW) is a key waste-to-energy strategy, yet daily biogas yield is often challenging to sustain, partly due to a limited understanding of the internal methanogens and their functional divergence. Here, we investigated seven full-scale mesophilic FW-AD systems distributed across China along a broad latitudinal gradient (>2800 km), linking methane production variations (0.38–2.11 m^3^/m^3^•d^−1^) with the phylogenetic distributions of methanogens and their methanogenic genes. We found that hydrogenotrophic and aceticlastic pathways were ubiquitous, whereas methylotrophic methanogenesis showed regional enrichment in warmer regions, reflecting persistent influences of climate-associated upstream conditions on downstream methanogenic communities. Gene-level phylogeny of methanogenesis-related alleles, rather than species-level phylogeny, closely tracked biogas yield variation (Mantel’s *P* < .05) and showed consistently stronger associations than gene-level compositions (mean standardized total effect: 0.491 vs. 0.298, *P* < .01). Higher methane yields (1.61 vs. 0.61 m^3^/m^3^•d^−1^ in high- vs. low-performing systems, *P* < .01) were significantly associated with reduced Faith’s phylogenetic diversity (1.82 vs. 2.30, *P* < .01) and tighter clustering (mean pairwise phylogenetic distance: 0.25 vs. 0.30, *P* < .01) of methanogenic gene variants, suggesting that phylogenetic coherence may reflect ecological filtering favoring efficient methanogenesis, albeit at the expense of functional redundancy. These findings highlight gene-level trait phylogeny as a potential proxy for functional robustness, offering a framework for ecological design of AD microbiomes.

## Introduction

The rapid accumulation of food waste (FW) has emerged as a critical environmental issue globally, with recent estimates reaching 2.5 Gt per year, far exceeding the 1.3 Gt reported just a decade ago [1–3]. Anaerobic digestion (AD) has been widely recognized as a sustainable and promising technology to convert FW into biogas, reducing the environmental burden of waste disposal [4–6]. As biogas production becomes increasingly crucial for waste-to-energy strategies, optimizing FW-AD system performance has garnered growing attention [4, 7, 8]. Central to this optimization is the microbial community, which mediates the biochemical reactions underlying organic matter degradation and methanogenesis [9–11]. Understanding the ecological and functional dynamics of these microbial communities, and how they relate to system performance, is essential for engineering more efficient and robust AD microbiomes [4, 10].

Over the past decades, extensive studies have been conducted to characterize the microbial communities inhabiting AD systems [10, 12, 13]. These studies have revealed the importance of syntrophic interactions between fermentative bacteria and methanogenic archaea, which drive the multi-step anaerobic conversion of complex organic substrates into methane [10, 14]. Distinct methanogenic lineages such as *Methanosaeta, Methanobacterium*, and *Methanosarcina* each engage in specific pathways (aceticlastic, hydrogenotrophic, and methylotrophic) or display mixotrophic metabolism through multiple methanogenic pathways, thereby contributing to biogas production in the system [10, 15, 16]. Molecular approaches, including 16S rRNA gene and shotgun metagenomic sequencing, have greatly advanced our understanding of microbial diversity, community dynamics, and assembly processes in AD environments [11, 17–19]. However, most of these efforts have focused on taxonomic composition or broad functional profiling, leaving a critical knowledge gap in how key microbial functional traits related to methane production are structured and distributed across environmental gradients.

Microbial functional traits, defined as measurable features linked to microbial performance and ecosystem functioning [20, 21], provide mechanistic insights beyond taxonomic identity and are essential for understanding microbiomes in both natural and engineered systems [22, 23]. This trait-centric perspective has proved valuable in linking microbial community diversity to environmental gradients and ecosystem function [21, 24, 25]. Enabled by the high-throughput sequencing revolution, microbial functional traits can be inferred from genomes and identified across multiple biological levels, including gene, organism, guild, and community [20, 26]. In AD systems, methanogenesis-related traits represent key functional axes, encompassing distinct methanogenic pathways (hydrogenotrophic, aceticlastic, and methylotrophic) and the central energy-conserving modules shared among them [27, 28]. These pathways differ in substrate affinities, metabolic intermediates, thermodynamic constraints, and energy yields, making their prevalence highly sensitive to environmental conditions such as ammonia toxicity, organic loading, pH, and temperature [29–32]. However, it remains unclear how external climatic factors and internal reactor operating conditions jointly shape the distribution of methanogenic pathways across large spatial scales.

Many functional genes involved in methanogenic pathways encode essential energy-conserving reactions and are therefore evolutionarily conserved under strong functional and thermodynamic constraints [28, 33, 34]. Such conservation reflects purifying selection, under which neutral and deleterious mutations are preferentially removed, leaving phylogenetic divergence that is more likely to reflect functionally meaningful variation than neutral drift. As a result, gene-level phylogenies provide a potential means to represent functional differentiation among homologous methanogenic genes across microbial taxa. Recent advances in metagenomic tools now enable the reconstruction of full-length gene sequences from complex environmental samples, allowing for more robust and accurate phylogenetic analyses [26, 35–37]. Tools such as REMIRGE, when combined with comprehensive reference databases (e.g. MCycDB, SCycDB, and NCycDB), can reassemble gene sequence clusters (GSCs) and generate reliable phylogenies [36]. Such approaches have been successfully applied in natural ecosystems such as deep-sea cold seeps, revealing how the phylogenetic diversity (PD) and evolution of functional genes relate to ecological processes and environmental filtering [35, 36]. Besides, the phylogenetic divergence of functional traits holds theoretical potential for indicating functional redundancy or specialization [38, 39], offering a new lens on ecosystem stability amid environmental change. However, the phylogenetic structure of functional genes and their ecological relevance remain largely unexplored in the context of engineered systems. In particular, it remains unknown whether and how the phylogenetic organization of methanogenic genes influences biogas production performance.

In this study, we investigated the compositional and phylogenetic divergence of methanogenesis-related genes across seven geographically distributed full-scale FW-AD systems in China. By integrating shotgun metagenomic data with environmental variables and gas production metrics, we sought to provide a trait-based and phylogenetically informed understanding of the methanogenic community structure and function within engineered AD ecosystems. Special emphasis was placed on the gene-level phylogenetic organization, a largely overlooked dimension in AD research. Grounded in this framework, we hypothesized that: (i) methanogenic functional traits and associated microbial communities exhibit systematic spatial differentiation across full-scale FW-AD systems; (ii) climatic and operational factors jointly shape the distribution of methanogenic pathways and the phylogenetic structure of methanogenic genes; and (iii) the gene-level phylogenetic organization of methanogenic traits reflects environmental filtering that favors functionally efficient configurations and is thereby linked to biogas production performance.

## Materials and methods

### Anaerobic digestion sample collection and metadata acquisition

AD samples were collected from seven full-scale FW-AD reactors across distinct geographic regions in China, spanning from the north to the south (Supplemental Fig. S1). These sites included Beijing (BJ; N40°2′58″, E116°6′31″), Qinhuangdao in Hebei Province (QH; N40°0′17″, E119°37′59″), and Qiqihar in Heilongjiang Province (QQ; N47°10′0″, E124°1′1″) in the north; and Changsha in Hunan Province (CS; N28°15′31″, E113°1′36″), Jingzhou in Hubei Province (JZ; N30°19′58″, E112°20′48″), Wenzhou in Zhejiang Province (WZ; N27°30′44″, E120°37′53″), and Foshan in Guangdong Province (FS; N22°59′52″, E113°0′6″) in the south. All reactors adopted comparable pre-treatment and operational processes (two-phase AD system) to minimize methodological variability, and detailed descriptions of these facilities have been reported previously [40].

Sample collection began in May 2022 and continued through September 2022, corresponding to the summer period. During this time, samples were collected continuously at 14-day intervals. Due to unavoidable circumstances at specific sites, the sampling interval was occasionally shortened, but the interval between two consecutive sampling events was never <7 days. Efforts were made to ensure that each site was sampled at least once per month, and a minimum of nine sampling events were conducted per site throughout the sampling period. Samples included both influent and in-reactor sludge from AD reactors. In total, 128 samples were obtained across the seven sites, comprising 64 influent samples and 64 in-reactor samples, with detailed sampling dates for each sample provided in Supplemental Table S1. Simultaneously, *in situ* operational parameters were recorded for each AD reactor based on real-time monitoring data from the facilities, including pH, temperature, daily gas production (GP, m^3^/d), methane content (MC, %), and daily feed volume (m^3^/d). In addition, local climatic variables, including 2-meter monthly mean air temperature (T2M), wind speed (WS2M), relative humidity (RH2M), and corrected precipitation (PRECTOTCORR), were retrieved for each site from the NASA POWER database using the R package nasapower [41].

### Analysis of physicochemical properties

Samples were transported to the laboratory under frozen conditions and stored at −40°C until further analysis. Physicochemical properties were measured for all 128 samples, including pH, salinity (‰), total nitrogen (TN, mg/l), ammonia nitrogen (NH_3_-N, mg/l), total solids (TS, %), soluble chemical oxygen demand (SCOD, mg/l), soluble carbohydrate (S-carbohydrate, g/l), and soluble protein (S-protein, μg/ml). Before physicochemical measurements, 50 ml of each homogenized sample was transferred into a centrifuge tube for pretreatment. The samples were centrifuged at 11 000 rpm for 30 min, and the supernatant was transferred to a new tube. This step was repeated 1–2 times until no or minimal precipitate remained. The resulting supernatant was sequentially filtered through 0.45 μm and 0.22 μm membrane filters. The final filtrate was stored at −20°C for subsequent physicochemical analyses.

An aliquot of 5 ml of the homogenized sample was used to determine TS following standard methods [42]. Salinity was assessed using a digital salinometer (PAL-06S, ATAGO, Japan), and pH was measured using a pH meter (PB-10, Sartorius, Germany). SCOD, TN, and NH_3_-N were measured using HACH reagent kits (HACH, USA) following the protocols described in our previous study [17]. S-carbohydrate was measured via the anthrone-sulfuric acid method with glucose standards [43], whereas S-protein was determined using Modified BCA Protein Assay Kit (Sangon Biotech, China) with a Microplate spectrophotometer (Multiskan FC, Thermo Scientific, USA).

### Evaluation of gas production performance

In addition to the *in situ* monitored indicators GP and MC, three additional indicators, namely methane production (MP, m^3^/d), average methane production (AMP, m^3^/m^3^•d^−1^), and average gas production (AGP, m^3^/m^3^•d^−1^), were used to assess the gas production performance of AD systems comprehensively. Among them, AMP and AGP account for differences in reactor design capacities (Supplemental Table S1), providing a normalized comparison across sites. The indicators were calculated as follows:


$$MP= GP\times MC$$

$$AGP=\frac{GP}{V_{ef}}$$

$$AMP=\frac{MP}{V_{ef}}$$

where ${V}_{ef}$ (m^3^) is the effective volume of the AD reactor.

### DNA extraction and 16S rRNA gene sequencing

The 64 AD sludge samples were used for total genomic DNA extraction. For each sample, 10 ml of well-mixed sludge was vacuum-filtered through a 0.45 μm membrane filter. The PowerSoil DNA Isolation Kit (MO BIO Laboratories, USA) was then used to extract total genomic DNA from the retained biomass on the filter, following the manufacturer’s protocol. DNA quality and quantity were assessed using a NanoDrop 2000 spectrophotometer (Nanodrop Technologies, USA). DNA samples with low purity (OD260/230 < 1.7) were desalted using ethanol and sodium acetate precipitation. More details are provided in Supplemental Materials and Methods.

The V4 region of prokaryotic 16S rRNA genes was amplified using a modified universal primer pair 515F and 806R [44], with sample-specific barcodes added to the 5′ ends. The polymerase chain reaction amplification was followed by purification via gel electrophoresis and quantification using a Nanodrop 2000 spectrophotometer (Nanodrop Technologies, USA). The purified amplicons were then subjected to paired-end sequencing (2 × 250 bp) on the NovaSeq System (Illumina) at Biozeron Biotechnology Co., Ltd (Shanghai, China). The raw sequence data were preprocessed using our in-house amplicon data analysis pipeline (https://dmap.denglab.org.cn) [45, 46], following the same procedures as in our previous study [47]. High-quality sequences were subsequently processed with Unoise3 [48, 49] under default parameters (minimum sequence abundance = 8; mapping threshold = 0.97) to generate Zero-radius operational taxa units (ZOTUs) and representative sequences. The resampled ZOTU table was used for subsequent biostatistical analysis (more details in Supplemental Materials and Methods).

### Shotgun metagenomic sequencing and analyses

Qualified genomic DNA from 64 AD sludge samples was subjected to paired-end (2 × 150 bp) shotgun metagenomic sequencing using the NovaSeq System (Illumina) at Biozeron Biotechnology Co., Ltd (Shanghai, China). For each sample, a minimum of 10 GB of raw sequencing data was generated. Duplicate reads were first removed using FastUniq (version 1.1) [50], followed by quality control with Trimmomatic (version 0.39) [51] to remove adapters and trim low-quality bases using the following parameters: SLIDINGWINDOW:4:20 LEADING:3 TRAILING:3 MINLEN:50 ILLUMINACLIP:TruSeq2-PE.fa:2:30:10:1:true. The quality of the cleaned reads was assessed with FastQC (version 0.11.7), and summarized across samples using MultiQC (version 1.16) [52].

To reconstruct full-length sequences of functional genes involved in methanogenesis, we employed Reprogrammed EMIRGE (REMIRGE) [36, 53] across all 64 samples using a co-assembly strategy. Specifically, a reference nucleotide sequence dataset comprising 585 225 sequences corresponding to 273 methane cycling genes was curated by retrieving the nucleotide counterparts of amino acid sequences from the MCycDB database [27] via NCBI. To enrich for full-length gene sequences, we applied a two-step length filtering criterion: (i) only genes with a maximum-to-minimum sequence length ratio > 1.25 were filtered; (ii) for each such gene, only sequences longer than the mean sequence length were retained. After filtering, 333 104 sequences remained.

Using these sequences, we built a Bowtie2-indexed reference database via REMIRGE’s make_my_db.py script with parameters -m 0 -M 100000 to retain all input sequences. Based on this reference, REMIRGE was run with default settings to reconstruct GSCs across all samples. The final output from the 40th iteration of the expectation–maximization algorithm was processed using abundance_calculate.py and normalization.py scripts to generate normalized abundance tables for each functional gene, accounting for both library size and gene length. These abundance tables were used for downstream statistical analyses. In addition, for each gene, multiple sequence alignments of reconstructed GSCs were performed using MAFFT (version 7.310) [54] in automatic mode, and corresponding phylogenetic trees were constructed using FastTree (version 2.1.3) [55] for subsequent phylogenetic analyses.

### Statistical analyses

*β*-diversity analyses of both microbial communities and functional traits were conducted in R (version 4.2.1), with community analyses based on the resampled ZOTU table and without additional filtering or statistical transformation. Bray–Curtis dissimilarities were calculated using the “vegan” package (version 2.6.4) [56], whereas unweighted UniFrac distances were computed via the “GUniFrac” package (version 1.8) [57, 58]. Principal coordinate analysis (PCoA) was performed with “vegan” [56], and dissimilarities in community and functional composition across sampling sites were tested using permutational multivariate analysis of variance (PERMANOVA). Variation partitioning analysis (VPA) was also implemented in “vegan” [56] to disentangle the relative contributions of geographic and environmental factors to observed compositional variation in methanogenic function. Multiple regression on matrices (MRM) analysis and partial Mantel tests were carried out using the “ecodist” package (version 2.1.3) [59–61] to explore the relationships between community composition, functional composition, gene-specific GSC profiles and different factors. Pairwise geographic distances among sites were computed using the “geosphere” package (version 1.5–18) [62]. All environmental variables were standardized to a zero mean and unit variance using “vegan”, and Euclidean distance matrices were subsequently derived. *α*-diversity indices were computed using the statistical tools embedded in the in-house amplicon sequencing analysis platform (https://dmap.denglab.org.cn) [45, 46]. For gene-level PD analyses, Faith’s PD and mean pairwise phylogenetic distance (MPD) were calculated using the “picante” package (version 1.8) (more details in Supplemental Materials and Methods). Lower MPD values were interpreted as indicating tighter phylogenetic clustering of gene variants. Correlations among environmental factors were assessed using Spearman’s rank correlation in the “Hmisc” package (version 5.1-1). The base “stat” package (version 4.2.1) was used for other correlation analysis and regression analysis. All *P* values from multiple hypothesis tests were adjusted using the false discovery rate (FDR) method [63]. To examine the complex interactions among community structure, functional composition, gene-specific phylogenetic traits, environmental factors, and gas production performance, structural equation modeling (SEM) was performed using IBM SPSS Amos (version 24.0.0, IBM Corp., Armonk, NY, USA). Model fit was evaluated based on the comparative fit index (CFI), root mean square error of approximation (RMSEA), and the probability of close fit (PCLOSE), using bootstrapped estimates. The model demonstrated a strong fit with CFI > 0.99, RMSEA <0.05, and PCLOSE >0.05. Phylogenetic trees were visualized using iTOL [64], and all other visualizations were generated in R.

## Results

### Operational conditions and performance across full-scale anaerobic digestion systems

All samples in this study were derived from two-phase, mesophilic, full-scale FW-AD systems operating under comparable engineering designs and located in seven cities across China (BJ, Beijing; CS, Changsha; FS, Foshan; QQ, Qiqihar; QH, Qinhuangdao; WZ, Wenzhou; and JZ, Jingzhou). In-reactor operating temperatures ranged from 37.41 ± 0.03°C (JZ) to 41.40 ± 0.57°C (WZ), all falling within the mesophilic operational window. Despite this technological consistency, substantial variation in both influent characteristics and in-reactor conditions was observed across the seven geographically distributed facilities (Supplemental Fig. S3). All systems maintained stable operational status and continuous biogas output (Supplemental Fig. S4), while environmental and performance parameters showed moderate temporal fluctuations during the sampling period (Supplemental Fig. S5). The average biogas production ranged from 0.64 ± 0.07 m^3^/m^3^•d^−1^ (WZ) to 4.26 ± 0.18 m^3^/m^3^•d^−1^ (BJ), whereas AMP varied more than five-fold, from 0.38 ± 0.05 m^3^/m^3^•d^−1^ (WZ) to 2.11 ± 0.28 m^3^/m^3^•d^−1^ (BJ). These disparities reflected the distinct system efficiencies and underlined the need to examine microbial and functional trait-level drivers underlying methane yield variation across engineered AD reactors.

### Community structure and associated environmental drivers

**Figure 1:**
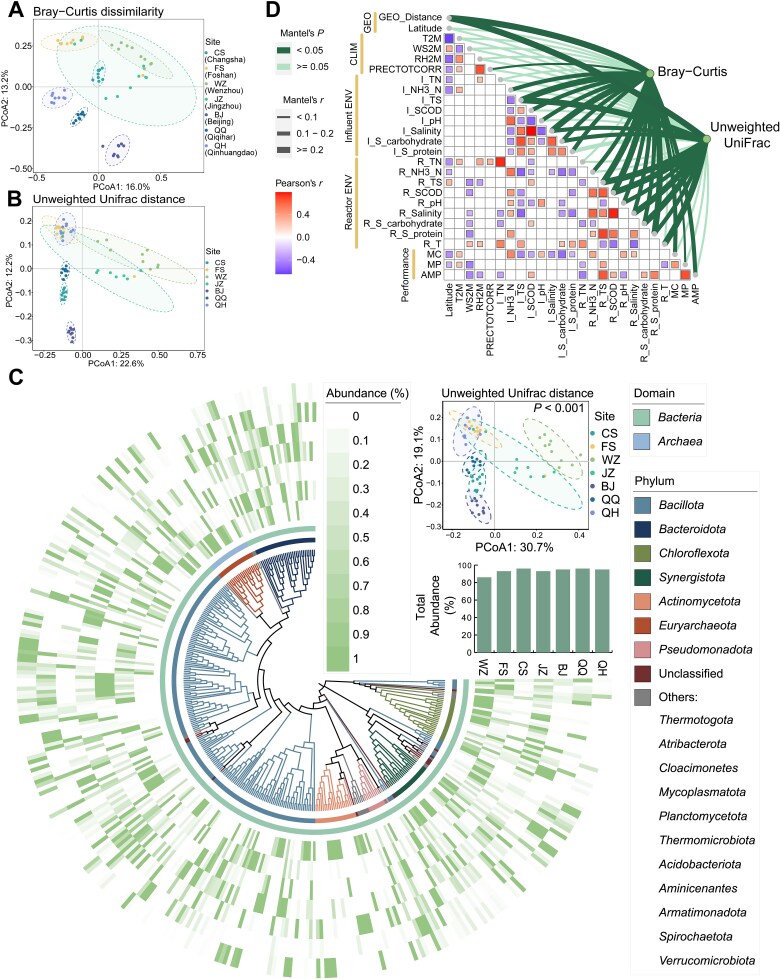
Regional variation in prokaryotic community structure and associated environmental drivers in AD systems. (A, B) Taxonomic (A) and phylogenetic (B) variations in prokaryotic community structure. (C) Phylogenetic and taxonomic distribution of abundant ZOTUs. The outermost heatmap represents the relative abundance of each ZOTU across different sites, with bar plots at the end of the heatmap indicating total relative abundance. The two middle rings denote the taxonomy of each ZOTU at the domain and phylum levels, respectively. Only the eight most diverse phyla are shown, while less diverse phyla are grouped into “Others.” The PCoA plot in the upper right corner illustrates the phylogenetic structural variation of abundant ZOTUs across different sites (*P* value obtained from PERMANOVA). (D) Partial Mantel tests (geographic distance-corrected) correlating taxonomic (Bray–Curtis dissimilarity) and phylogenetic (unweighted UniFrac distance) community variation with environmental factors (including local climate variables [CLIM], influent/in-reactor physicochemical properties [Influent/Reactor ENV]) and gas production performance (Performance). The thickness of the lines reflects the strength of the correlation. The color of the lines reflects the statistical significance of this relationship. The Spearman’s rank correlation coefficients between pairwise factors are expressed as color gradients, and only significantly correlated relationships are shown in colored boxes.

A total of 3488 taxa (ZOTUs) were obtained from seven FW-AD facilities by using 16S rRNA gene amplicon sequencing. *β*-diversity analysis revealed significant taxonomic variation (based on Bray–Curtis dissimilarity) and phylogenetic variation (based on unweighted UniFrac distance) among different facilities in both the overall prokaryotic community (Fig. 1A, B, Supplemental Table S2) and the abundant taxa (Fig. 1C). The abundant taxa (the average relative abundance in at least one site >0.1%; *n* = 337) accounted for more than 80% of the total abundance (Fig. 1C).

SEMs revealed that both influent characteristics and in-reactor environmental factors significantly influenced taxonomic composition, which in turn exerted a strong direct effect on gas production (|*β*| = 0.397–0.485; Supplemental Fig. S6). Partial Mantel tests for each deterministic factor further disentangled their individual role in community assembly (Fig. 1D). After controlling for the effect of geographic distance, most environmental factors within AD reactors exhibited significant correlations with community composition. Specifically, pH, salinity, and substrate-related factors such as soluble protein (S-protein), soluble carbohydrate (S-carbohydrate), SCOD, and total solids (TS) were significantly associated with both taxonomic and phylogenetic variation. Temperature within AD reactors influenced only the phylogenetic structure of the community. Regarding influent characteristics, TS, S-carbohydrate, and salinity, were strongly correlated with community composition. Other influent characteristics, such as pH, ammonium nitrogen (NH_3_-N), SCOD, and S-protein, exhibited significant associations with taxonomic composition but not with phylogenetic variation (Fig. 1D). Similarly, the MRM analysis further confirmed that substrate-related factors, including influent/in-reactor TS, SCOD, and S-carbohydrate within AD reactors, as well as influent/in-reactor salinity, were all significantly associated with community composition (Supplemental Table S4). These results suggested that phylogenetic and taxonomic diversity of the entire microbial community might have different environmental drivers in AD reactors, but substrate-related factors (e.g. TS, protein-related indexes), along with pH and salinity, consistently act as key drivers of both community dimensions. Moreover, these factors exert taxon-specific and directionally heterogeneous effects, with different microbial taxa exhibiting contrasting responses along environmental gradients (Supplemental Fig. S7).

### Functional gene profiles of methanogenesis

A total of 120 methanogenesis-related functional genes were reconstructed across all samples, yielding 3383 full-length GSCs, also referred to as alleles, which represent distinct gene variants defined at 97% sequence identity. Across the four major methanogenic pathways, hydrogenotrophic, aceticlastic, methylotrophic, and central methanogenesis, we reconstructed 13 (370 GSCs), 12 (878 GSCs), 15 (297 GSCs), and 80 (1838 GSCs) functional genes, respectively. Among the 114 methanogenic genes represented by more than one GSC, 97 were detected across all sampling sites, and 49 were consistently present in all 64 AD sludge samples (Supplemental Dataset S1).

**Figure 2:**
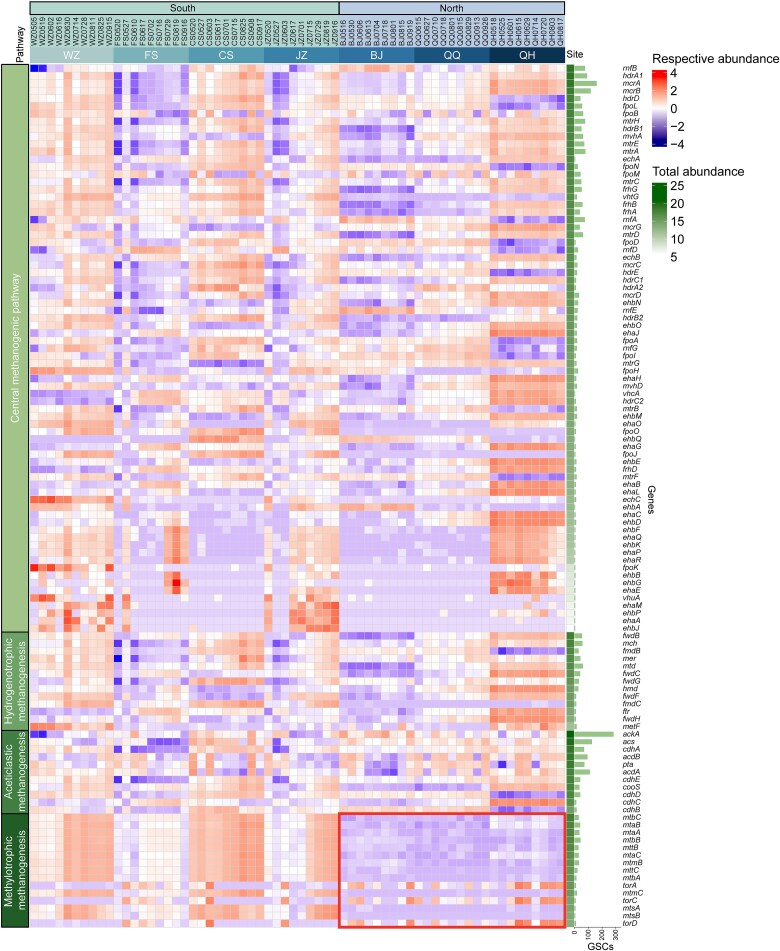
Abundance and diversity of methanogenesis functional genes. The heatmap displays the respective abundance (log-transformed and row-standardized to a mean of 0 and unit variance) of each functional gene across samples. Blocks on the right indicate the total abundance of each gene, arranged from highest to lowest within each pathway. The diversity of each functional gene, measured as the number of GSCs, is illustrated as a bar chart on the rightmost. The sampling site information for each sample is annotated at the top, and samples are grouped based on northern and southern geographic locations. The methanogenic pathway associated with each gene is annotated on the left. The central methanogenic pathway refers to gene families shared across hydrogenotrophic, aceticlastic, and methylotrophic methanogenesis that encode the conserved core steps of methane formation, following the MCycDB classification. Only genes with 2 or more GSCs are shown.

Among the three different substrate types of methanogenesis pathways, aceticlastic methanogenesis exhibited the highest total abundances (mean: 440 608), followed by methylotrophic (90 348) and hydrogenotrophic methanogenesis (83 905) (Fig. 2). Similarly, functional gene richness was also significantly higher in aceticlastic methanogenesis (mean: 80 GSCs) compared to methylotrophic (20 GSCs) and hydrogenotrophic (28 GSCs) methanogenesis. Genes associated with aceticlastic and hydrogenotrophic methanogenesis were detected in an average of 99.6% and 91.6% of the samples, respectively. In comparison, those related to methylotrophic methanogenesis were present in only 74.3% of the samples (Supplemental Dataset S1). Besides, functional genes associated with methylotrophic methanogenesis exhibited a distinct latitudinal pattern, with significantly higher abundance at southern sites than northern sites (Fig. 2). Within the central methanogenesis pathway, the *rnfB* gene, encoding NAD^+^ oxidoreductase, exhibited the highest total abundance, whereas the *mcrA* gene, encoding Methyl-coenzyme M reductase, displayed the greatest diversity (Fig. 2). In the aceticlastic methanogenesis pathway, the *ackA* gene, encoding acetate kinase, exhibited both the highest abundance and diversity (Fig. 2).

### Environmental drivers of methanogenic function

**Figure 3:**
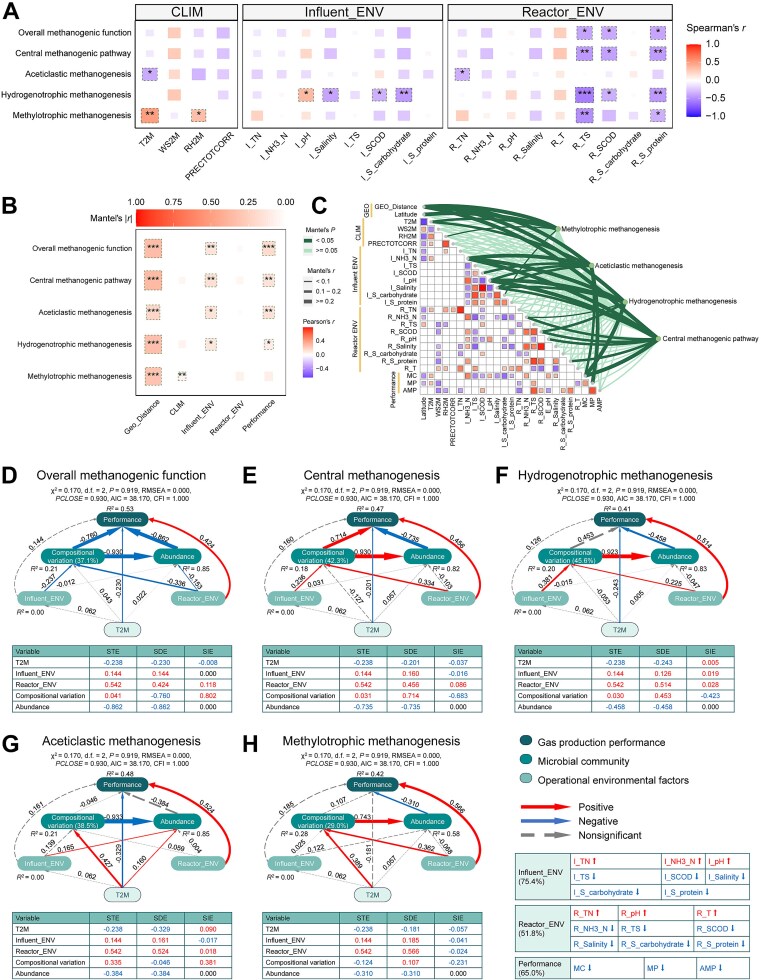
Environmental drivers of methanogenic functional composition in AD microbiomes across regions. (A) Spearman correlations between the abundance of methanogenic pathways and environmental factors. A color gradient and box size indicate correlation coefficients. *P* values were adjusted using the FDR method and significance levels are annotated within the boxes (*: *P* < .05, **: *P* < .01, ***: *P* < .001). (B, C) Partial Mantel tests between the functional compositional variation (geographic distance-corrected) of methanogenic pathways and grouped factors (B) or individual factors (C). Functional compositional variation was quantified using Bray–Curtis dissimilarity based on pathway-level gene abundance profiles. (D–H) SEMs depicting the relationships between functional composition of the overall methanogenic function (D), central methanogenesis (E), hydrogenotrophic methanogenesis (F), aceticlastic methanogenesis (G), and methylotrophic methanogenesis (H), and environmental factors and gas production performance. Overall methanogenesis represents the combined functional composition of all methanogenesis-related genes across pathways. Functional composition, influent/in-reactor environmental factors, and overall performance are represented by the first principal coordinate (PC1) derived from Bray–Curtis-based PCoA. The explained proportions of each PC1 and its positive/negative correlations with associated variables are shown in the boxes at the bottom right. The numbers beside the arrows represent standardized path coefficients (*β*), and arrow thickness is proportional to the strength of the effect. *R*^2^ denotes the proportion of variance explained for each endogenous variable. Standardized total effects (STE), standardized direct effects (SDE), standardized indirect effects (SIE), and model fit indices are listed below each SEM panel.

To investigate the driving forces underlying the distribution patterns of methanogenic pathways, we analysed the correlations between pathway abundance and environmental factors (Fig. 3A). Except for aceticlastic methanogenesis, the abundances of the other three methanogenic pathways, as well as the overall methanogenic function, exhibited strong negative correlations with substrate-related factors within AD reactors, including TS, SCOD, and S-protein. This indicates that higher TS, SCOD, and S-protein were associated with lower relative abundances of these methanogenic functions. Additionally, climatic factors, such as monthly mean air temperature (T2M), showed significant positive and negative associations with methylotrophic and aceticlastic methanogenesis, respectively. Regression analyses further revealed that among all associated environmental factors, T2M exhibited the highest explanatory power for the variation in methylotrophic and aceticlastic methanogenesis pathway abundances (*R*^2^ = 0.25 and 0.17, respectively; Supplemental Fig. S8A and F). For hydrogenotrophic methanogenesis, TS and SCOD within AD reactors were the most influential factors (Supplemental Fig. S8M and N).

The functional composition of nearly all methanogenic pathways exhibited significant variation across the seven sites (Supplemental Table S5). VPA showed that geographic distance and environmental factors together explained 33.1% of the variation in overall methanogenic functional composition, with environmental factors contributing more than geographic distance (Supplemental Fig. S9A). After controlling for geographic distance, the partial Mantel tests revealed that the functional compositions of overall, central, aceticlastic, and hydrogenotrophic methanogenesis were significantly associated with influent characteristics, especially NH_3_-N, pH, and salinity (Fig. 3B, C, Supplemental Fig. S9B). In contrast, the functional composition of methylotrophic methanogenesis was significantly correlated with climatic factors (Fig. 3B). T2M was strongly associated with the functional composition of both methylotrophic and aceticlastic methanogenesis (Fig. 3C). In-reactor factors such as NH_3_-N, pH, SCOD, and S-protein were significantly linked to the functional composition of overall, central, and hydrogenotrophic methanogenesis (Fig. 3C, Supplemental Fig. S9B). Furthermore, except for methylotrophic methanogenesis, functional compositions of other methanogenic pathways were significantly associated with gas production performance (Fig. 3C, Supplemental Fig. S9B).

SEM was further employed to quantify the direct and indirect effects of environmental factors and methanogenic functional traits on gas production performance (Fig. 3D–H, Supplemental Fig. S10). The functional composition and abundance of overall and central methanogenesis exerted strong direct effects on gas production performance (Fig. 3D, E, Supplemental Fig. S10A, B), whereas the effects of hydrogenotrophic, aceticlastic, and methylotrophic pathways were comparatively weak or non-significant (Fig. 3F–H, Supplemental Fig. S10C–E). In general, pathway abundance exhibited stronger direct and total effects on gas production performance than functional composition (Fig. 3D–H, Supplemental Fig. S10).

Influent and in-reactor factors, particularly the latter, had significant direct effects on the functional composition of most methanogenic pathways and indirectly influenced their abundance (Fig. 3D–F, H). Only the abundance of aceticlastic methanogenesis was directly affected by influent factors (Fig. 3G). Overall, in-reactor factors exerted a greater total effect on gas production performance than influent factors, primarily through direct effects (Fig. 3D–H, Supplemental Fig. S10). Their indirect effects were primarily mediated by shaping the functional composition and abundance of overall and central methanogenesis. Additionally, T2M exhibited significant direct effects on gas production performance (Fig. 3D–G), whereas its indirect effects were restricted to aceticlastic and methylotrophic pathways (Supplemental Fig. S10D and E).

### Linking methanogenesis genes to methane production

The compositional and phylogenetic features of GSCs in methanogenesis genes were further investigated to determine how they affect gas production performance. A total of 25.4% to 43.0% of functional genes showed significant correlations between their abundance and gas production performance (Supplemental Fig. S11A). Among these, 5.3% to 19.3% of genes exhibited positive correlations, particularly with methane contents (MC). These included genes from the central methanogenesis pathway (e.g. *rnfBD, echAB, ehaBGHJ, hdrC1C2, ehbQ, vhcA*), hydrogenotrophic pathway (e.g. *fwdBC, ftr, mtd*), and aceticlastic pathway (e.g. *ackA, acs, cooS, cdhBC*). Several environmental factors, including influent pH, salinity, SCOD, and S-carbohydrate, as well as in-reactor TS, SCOD, and S-protein, exhibited strong associations with gene abundance.

**Figure 4:**
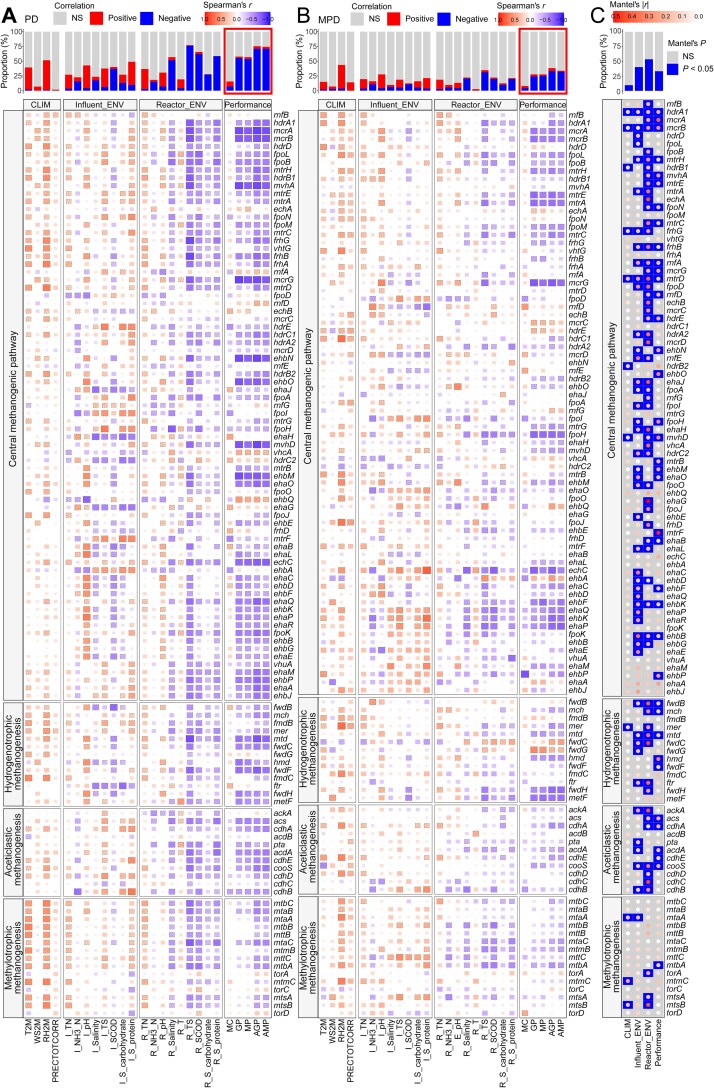
Phylogenetic features of methanogenesis functional genes in relation to gas production performance. (A, B) Spearman correlations between the PD of each functional gene (measured by Faith’s PD in A and MPD in B) and environmental factors, and gas production performance. A color gradient and box size indicate correlation coefficients. Statistically significant correlations (*P* < .05) were highlighted with dashed boxes. Bar plots on the top indicate the proportion of functional genes significantly positively or negatively correlated with each factor. *P* values were adjusted using the FDR method. (C) Partial Mantel tests between the phylogenetic variation (geographic distance-corrected) of each functional gene and different categories of factors. Mantel’s |*r*| values are visualized using color gradients, with significant correlations highlighted in blue. Bar plots on the top indicate the proportion of genes significantly associated with each factor category.

The diversity of GSCs was generally significantly correlated with gas production performance, particularly MP, AMP, GP, and AGP (Fig. 4A, B, Supplemental Fig. S11B). Both richness (Supplemental Fig. S11B) and PD (Faith’s PD and MPD; Fig. 4A and B) of a considerable number of genes showed negative associations with gas production performance. Consistently, the seven AD systems could be stratified into two groups with significantly different gas production performance (Supplemental Fig. S4, Supplemental Table S6): a high-performing group (BJ, CS, and FS; mean AMP: 1.61 m^3^/m^3^•d^−1^) and a low-performing group (QQ, QH, WZ, and JZ; mean AMP: 0.61 m^3^/m^3^•d^−1^). The high-performing group displayed significantly lower Faith’s PD (1.82 vs. 2.30, *P* < .01) and MPD (0.25 vs. 0.30, *P* < .01) compared to the low-performing group (Supplemental Table S6), further supporting the notion that AD systems with fewer and more closely related methanogenic gene variants tended to achieve higher methane yields. GSC diversity was more strongly associated with in-reactor environmental factors, especially substrate-related factors such as TS, SCOD, and S-protein, as well as salinity.

More than 25% of the functional genes exhibited significant correlations between their GSC compositional (Supplemental Fig. S12A) or phylogenetic (Fig. 4C) structure (*β*-diversity) and gas production performance. GSC compositional variation was more frequently associated with influent environmental factors (especially pH and salinity) than with in-reactor factors, although in-reactor SCOD and S-protein were also significantly linked to the GSC composition of many genes (Supplemental Fig. S12A and B). In contrast, the phylogenetic structure of genes was more strongly associated with in-reactor factors, particularly substrate-related factors such as TS, SCOD, and S-protein (Fig. 4C, Supplemental Fig. S12C).

**Figure 5:**
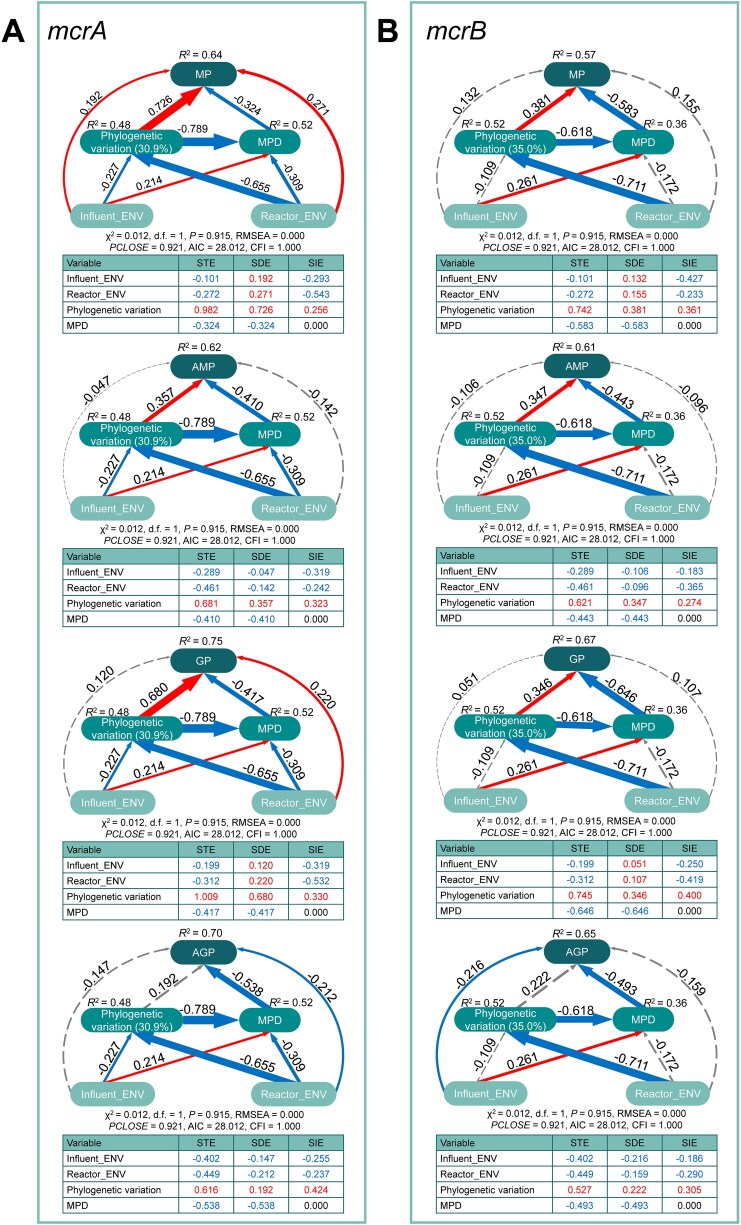
SEMs depicting the relationships between phylogenetic variation of two representative genes, *mcrA* (A) and *mcrB* (B), from the central methanogenesis pathway, and environmental factors, and gas production performance. Phylogenetic variation is represented by PC1 derived from unweighted UniFrac-based PCoA.

To further disentangle the direct and indirect effects among GSC compositional/phylogenetic variation, environmental factors, and gas production performance, we constructed a series of SEMs based on representative methanogenesis genes (*mcrA, mcrB, ackA, fwdB*, and *mtbC*) (Fig. 5, Supplemental Fig. S13, Supplemental Fig. S14). These representative genes encode key enzymes catalyzing pathway-defining reactions and were selected because they are consistently dominant within each methanogenic pathway (Fig. 2). Except for methylotrophic methanogenesis, representative genes from other methanogenic pathways exhibited significant direct effects of GSC composition on gas production performance (Supplemental Fig. S13). The richness of *mcrAB* from the central methanogenesis pathway and *fwdB* from the hydrogenotrophic pathway had strong negative direct effects on gas production performance (Supplemental Fig. S13A, B, D). In addition, the richness of most representative genes was primarily influenced by in-reactor factors (Supplemental Fig. S13A–C, E), whereas GSC composition was more strongly affected by influent factors (Supplemental Fig. S13A–C).

All representative genes showed significant direct effects of their phylogenetic features (i.e. the phylogenetic variation and MPD) on gas production performance (Fig. 5A, B; Supplemental Fig. S14). Furthermore, the total and direct effects of phylogenetic variation on gas production performance were generally stronger than those of compositional variation (Supplemental Fig. S15, Table S7) and were predominantly driven by in-reactor factors (Fig. 5A, B; Supplemental Fig. S14). MPD consistently exhibited negative effects on all performance indicators related to gas production.

## Discussion

Microbial communities in AD reactors are central to process efficiency [9–11], yet the functional traits that mediate microbial contributions to system performance, particularly those beyond taxonomic identity, remain poorly understood. Most studies to date have focused on taxonomic composition or general functional profile [11–13, 17–19, 65, 66], without fully addressing how the ecological structure of functional traits, particularly their phylogenetic organization, relates to reactor performance. This gap is especially relevant because microbial functional traits, such as methanogenic potential, are shaped not only by environmental selection but also by evolutionary constraints [28, 33, 34, 67]. In this study, we conducted a systematic national scale measurement of full-scale FW-AD systems and revealed pronounced variations in both the microbial community composition and methanogenic functions (Figs 1, 2).

Methanogenic pathways, which represent the terminal reactions in the AD system, are not static but are dynamically shaped by environmental and physicochemical conditions [29, 32, 68, 69]. In our study, both hydrogenotrophic and aceticlastic methanogenesis were consistently detected across all seven geographically distributed mesophilic FW-AD systems (Fig. 2, Supplemental Dataset S1), suggesting that these two routes form a metabolic backbone of methane production under mesophilic conditions [29, 32, 70]. In contrast, the methylotrophic methanogenesis pathway was significantly enriched in southern Chinese cities (Fig. 2), revealing a distinct pattern of functional biogeography. This spatial pattern is particularly intriguing given the pronounced climatic and dietary differences between northern and southern regions of China, suggesting an underlying influence of regional environmental or socio-ecological factors [71]. For example, the south typically experiences higher temperatures, greater humidity, and more plant- and fermentation-rich diets, which may increase the input of methylated compounds into the system.

The monthly mean air temperature (T2M) during the sampling month was further identified as the strongest explanatory variable for methylotrophic and aceticlastic pathway abundance (Supplemental Fig. S8A and F), with significant direct and indirect effects on methane yield (Fig. 3D–H, Supplemental Fig. S10). This finding challenges the conventional view of full-scale AD reactors as tightly insulated systems buffered from external climate influences, raising important questions about the pathways through which T2M exerts influence. Two non-mutually exclusive mechanisms may explain this pattern. First, T2M may serve as a proxy for unmeasured environmental heterogeneity in the influent, such as regionally structured differences in FW composition, storage conditions, or preprocessing practices. Second, T2M could influence the microbiome of the influent itself, particularly during the upstream hydrolysis and acidogenesis phases, which lack active temperature regulation in the two-phase AD systems studied [72–74]. This is consistent with recent evidence showing that historical or upstream conditions can exert persistent influences on downstream microbial community structure and function [47, 75–78], a phenomenon commonly referred to as legacy effects [79–81]. These findings underscore the ecological permeability of engineered systems to external conditions and highlight the importance of considering regional climate and feedstock variability in AD system design and optimization, particularly in decentralized or climate-sensitive operations.

In linking methanogenic function to system performance, our results demonstrated that methane production in full-scale mesophilic AD systems is more strongly associated with the quantitative strength of overall and core methanogenic functions than with the prevalence of any single methanogenic pathway (Fig. 3D–H, Supplemental Fig. S10). Moreover, although in-reactor factors exhibited a stronger overall influence on biogas output than influent characteristics, their direct effects on methanogenic pathway abundance were limited to overall methanogenesis (Fig. 3D, Supplemental Fig. S10A). This highlights the ecological importance of pathway centrality and overall trait representation over the dominance of any single methanogenic strategy [29, 82, 83]. A substantial portion of the in-reactor influence on methane yield could not be accounted for by methanogenic pathway abundance alone, indicating the presence of additional and critical regulatory layers beyond gene-level functional potential. These included the structure and activity of syntrophic partners supplying key intermediates (e.g. H_2_, acetate), variations in hydrogen partial pressure or redox gradients, micronutrient availability (e.g. Ni, Co essential for Mcr complexes), and post-transcriptional or enzymatic regulation [84–87].

Beyond functional pathway composition, phylogenetic divergence represents a critical yet often underappreciated dimension of microbial functional traits. Our study reconstructed gene-level phylogenies for each methanogenesis-related functional gene based on GSCs, and found that AD systems characterized by lower PD and tighter clustering within methanogenic GSCs were associated with higher methane yields (Fig. 4A, B, Supplemental Table S6). Although a substantial proportion of methanogenesis-related genes, particularly core gene families such as *mcr* and *mtr*, showed significant negative associations between phylogenetic divergence and methane yields, some other genes showed no significant relationships. This heterogeneity likely reflects differences in evolutionary history, functional constraint, and intrinsic phylogenetic divergence among methanogenesis-related genes, with more conserved and functionally central genes tending to exhibit clearer performance-linked phylogenetic signals. Phylogenetically closer gene variants are likely to encode enzymes with similar catalytic properties [88–90]. Thus, reduced phylogenetic divergence within each methanogenic gene therefore indicates the dominance of evolutionarily similar functional variants, and when this pattern occurs across multiple genes, it can reduce functional heterogeneity along the pathway at the community level, potentially enhancing the overall efficiency of the methanogenic pathway. From an ecological perspective, this pattern suggests ecological filtering across the methanogenic pathway that filters out less efficient variants while retaining more catalytically efficient configurations. Future studies integrating paired influent and reactor metagenomes will be important for disentangling the relative roles of dispersal and environmental selection in shaping gene-level phylogenetic divergence in engineered microbial ecosystems. This will help clarify the extent to which the observed phylogenetic structure reflects upstream introduction of functional gene variants versus ecological filtering within reactors.

The phylogenetic divergence of methanogenic GSC, rather than species-level phylogeny, exhibited a significant correlation with system performance (Fig. 4C, Supplemental Table S3), particularly with AMP (Supplemental Fig. S12C). Moreover, the phylogenetic divergence also showed consistently stronger associations with reactor biogas output than GSC compositional variation (mean |STE|: 0.491 vs. 0.298, *P* < .01; Supplemental Fig. S15, Table S7). It suggests that the evolutionary structure embedded within individual functional genes captures ecologically and biochemically relevant trait variation that is not detectable through taxonomic or abundance-based metrics [87, 91]. Together, these insights highlight the ecological and engineering value of integrating gene-level phylogenetic metrics into microbial trait frameworks. From an operational perspective, monitoring the phylogenetic divergence of methanogenesis-related genes may help identify reactor communities dominated by evolutionarily coherent and potentially more efficient functional variants. Such gene-level phylogenetic metrics could complement conventional indicators based on taxonomic composition or functional gene abundance, providing an additional tool for evaluating and optimizing methane production in AD systems.

Another important ecological insight from incorporating gene-level phylogenetic divergence into microbial trait frameworks is that it may provide information about the degree of functional redundancy within microbial functional gene pools. According to phylogenetic niche conservatism, closely related lineages tend to retain similar functional traits and ecological niches due to evolutionary constraints [39]. A narrower phylogenetic divergence with lower MPD among GSCs implies that the function is maintained by closely related allelic variants, which lack the ecologically differentiated variants capable of substituting under stress and thus have lower resilience to environmental perturbations [26, 92, 93]. Consequently, phylogenetic divergence of functional gene variants may provide an indirect indication of microbial functional redundancy and stability. In this sense, our observation that lower MPD in methanogenesis-related genes correlates with higher methane yield suggests a tradeoff between performance and stability in engineered microbiomes, where tightly clustered functional alleles may maximize pathway efficiency at the potential cost of resilience to functional disruption under stress. Across diverse natural and engineered ecosystems, observations support a general ecological tradeoff between productivity or efficiency and stability [94–96], and our results provide a gene-level phylogenetic perspective for understanding how such a tradeoff may emerge in AD microbiomes. This perspective highlights that optimization of AD performance should balance methane production efficiency with the maintenance of functional robustness under environmental perturbations. These insights raise the possibility that system performance and functional stability may be modulated by the phylogenetic divergence of key metabolic traits. Future research across more diverse ecosystems is needed to test this hypothesis and to compare gene-level phylogenetic metrics with conventional stability proxies. Such efforts will help clarify the utility of phylogenetic divergence of traits as an indicator of functional robustness and advance our understanding of the ecological and engineering relevance of trait phylogeny in microbial communities.

## Supplementary Material

wrag083_Supplemental_Files

## Data Availability

The sequencing data have been deposited in the National Genomics Data Center (NGDC) BioProject database (https://ngdc.cncb.ac.cn/bioproject/) under the BioProject accession numbers PRJCA042418 and PRJCA045032. The 16S rRNA gene and shotgun metagenomic sequencing reads are available in the Genome Sequence Archive (GSA) under accession numbers CRA027678 and CRA029320. All processed datasets and associated metadata supporting the statistical analyses are publicly available at https://github.com/yedeng-lab/Phylogeny-of-Methanogenic-Genes-in-Anaerobic-Digestion, which also provides the scripts used for data analysis and visualization. The code is released under the MIT License and archived on Zenodo with a permanent DOI: https://doi.org/10.5281/zenodo.18832853.
