# Supplementary material for: Phylogenetic assembly of methanogenesis regulates methane yield in food-waste anaerobic digestion
Source: ISME J. 2026 Apr 11;20(1):wrag083. doi: 10.1093/ismejo/wrag083 (PMC13200284; doi:10.1093/ismejo/wrag083)
Supplement: wrag083_Supplemental_Files [file wrag083_supplemental_files.zip › Supplemental file.docx]

**Phylogenetic assembly of methanogenesis regulates methane yield in food-waste anaerobic digestion**

Bo Zhao ^1,2^, Xingsheng Yang ^1,2^, Kai Feng ^1,2^, Jie Wang ^3^, Mingqian Liu ^1,2^, Yingcheng Wang ^4^, Danrui Wang ^5^, Xi Peng ^1,2^, Qing He ^1,2^, Yanjuan Lu ^6^, Hassan Waseem ^7^, Shang Wang ^1,2^, Ye Deng ^1,2*^

^1^ State Key Laboratory of Regional Environment and Sustainability, Research Center for Eco-Environmental Sciences, Chinese Academy of Sciences, Beijing 100085, China

^2^ University of Chinese Academy of Sciences, Beijing 100049, China

^3^ State Key Laboratory of Biogeology and Environmental Geology, China University of Geosciences, Beijing 100053, China

^4^ Qinghai Provincial Key Laboratory of Restoration Ecology in Cold Regions, Northwest Institute of Plateau Biology, Chinese Academy of Sciences, Xining, China
^5^ Soil Ecology Lab, Jiangsu Collaborative Innovation Center for Solid Organic Waste Resource Utilization and Jiangsu Key Laboratory for Solid Organic Waste Utilization, Nanjing Agriculture University, Nanjing 210095, China

^6^ Fairyland Environmental Technology Co., Ltd, Beijing 100085, China

^7^ Department of Civil and Environmental Engineering, Carleton University, 1125 Colonel By Dr, Ottawa, ON K1S 5B6, Canada

^*^Corresponding authors:

Ye Deng

CAS Key Laboratory for Environmental Biotechnology, Research Center for Eco-Environmental Sciences, Chinese Academy of Sciences, Beijing 100085, China

18 Shuangqing Road, Haidian District, Beijing 100085, China.

Telephone: +86 (010) 6284 0082

Fax: +86 (010) 6284 0082

E-mail: yedeng@rcees.ac.cn

**Supplemental Materials and Methods**

**DNA extraction and desalting**

Total genomic DNA from the 64 anaerobic digester sludge samples was extracted using the PowerSoil DNA Isolation Kit (MO BIO Laboratories, USA). Specifically, 10 mL of homogenized sludge was filtered through a 0.45 μm membrane. The retained material on the membrane was carefully cut into small fragments together with the membrane and transferred to the bead-containing tubes provided with the kit. DNA extraction was then carried out in accordance with the manufacturer's instructions. The concentration and purity of extracted DNA were evaluated using a NanoDrop 2000 spectrophotometer (Nanodrop Technologies, USA). DNA samples with suboptimal purity (OD260/230 < 1.7) were subjected to desalting via ethanol and sodium acetate precipitation to remove residual contaminants. The desalting procedure was carried out as follows:

1. DNA was precipitated by adding 2.5 vol of pre-chilled 100% ethanol and 1:10 volume of 3 M sodium acetate (pH 5.2), followed by overnight incubation at -20°C.
2. The mixture was centrifuged at 13,000 x *g* for 30 minutes to pellet the DNA. The supernatant was carefully discarded, and the pellet was washed with 1 mL of 70% cold ethanol.
3. A second centrifugation was performed at 13,000 x *g* for 10 minutes, after which the ethanol was removed.
4. The DNA pellet was air-dried for approximately 30 minutes (avoiding over-drying).
5. Finally, the DNA was resuspended in nuclease free water for downstream applications.

**16S rRNA gene sequence processing**

For amplification of the prokaryotic 16S rRNA gene, the conserved V4 region was targeted using the universal primers 515F (5′-GTGYCAGCMGCCGCGGTAA-3′) and 806R (5′-GGACTACHVGGGTWTCTAAT-3′) [1]. PCR amplification and product purification followed protocols established in our earlier study [2]. The purified amplicons were quantified using a Nanodrop 2000 spectrophotometer (Nanodrop Technologies, USA), pooled in equimolar amounts, and used to construct sequencing libraries. High-throughput paired-end sequencing (2 × 250 bp) was then conducted on the NovaSeq System (Illumina) at Biozeron Biotechnology Co., Ltd (Shanghai, China).

Raw sequencing reads were initially demultiplexed based on their unique barcodes, followed by the trimming of both primers and barcodes. Paired-end reads were then assembled using FLASH [3], and the resulting merged sequences were subjected to quality filtering with Btrim [4], using an average quality score threshold of 20 and a minimum length of 140 bp. This process yielded 6,695,527 high-quality sequences, with per-sample counts ranging from 19,209 to 219,323. The Unoise3 pipeline [5, 6] was subsequently applied for generating Zero-radius operational taxa units (ZOTUs) and representative sequences under default parameters (minimum sequence abundance = 8; mapping threshold = 0.97). To evaluate sequencing sufficiency, rarefaction curves were generated (Supplemental Figure S2), confirming that the sequencing depth was adequate to capture the majority of ZOTUs in each sample. To mitigate differences in sequencing depth, the ZOTU table was rarefied to the lowest sequence count across all samples (19,097 reads per sample). This resampled ZOTU table was then used for downstream statistical analyses.

Taxonomic assignment of representative ZOTU sequences was conducted using the Ribosomal Database Project (RDP) classifier (version 2.14) [7] against the RDP training set No. 19. For phylogenetic inference, MAFFT [8] was employed to align representative sequences in automatic mode, and a phylogenetic tree was constructed using FastTree [9] based on the multiple sequence alignment.

**Calculation of phylogenetic diversity metrics**

Gene level phylogenetic diversity of methanogenesis related functional genes was quantified using Faith's phylogenetic diversity (PD) and the mean pairwise phylogenetic distance (MPD), as implemented in the R package picante (version 1.8) [10].

Faith's PD quantifies the phylogenetic breadth spanned by the gene variants detected in a sample. It is computed as the sum of the branch lengths of the subtree connecting all observed variants [11]:

$$Faith^{'}s PD= \sum_{i}^{n} l_{i}$$

Where *l_i_* is the length of branch 𝑖, and *n* is the set of branches required to connect the detected variants on the gene-level phylogenetic tree. This metric reflects how widely the functional gene variants are distributed across the evolutionary space represented by that gene.

MPD measures the average phylogenetic distance among variants within a sample [12]. In this study, MPD was calculated in an abundance weighted form [13]:

$$MPD= \frac{\sum_{i}^{n} \sum_{j}^{n} d_{ij}p_{i}p_{j}}{\sum_{i}^{n} \sum_{j}^{n} p_{i}p_{j}}, where i \neq j$$

Where *n* is the number of observed gene variants, *p_i_* and *p_j_* are the relative abundances of variants *i* and *j* respectively, and *d_ij_* is the cophenetic distance between them on the phylogenetic tree.

This formulation captures the degree of phylogenetic clustering while accounting for the dominance structure of gene variants within each sample. Abundance weighted MPD therefore incorporate both evolutionary relatedness and variant dominance. Lower MPD values indicate that dominant gene variants are more closely related, reflecting tighter phylogenetic clustering, whereas higher values indicate greater phylogenetic dispersion.

Phylogenetic trees were reconstructed separately for each functional gene based on gene sequence clusters (GSCs), and pairwise distances were derived from branch length information. All calculations followed the standard implementation of phylogenetic community metrics in R package *picante*.

**Definition of GSCs**

Because functional genes do not have a universally accepted sequence identity threshold for defining gene clusters, we treated the clustering cutoff as an operational rather than a strict biological boundary. In this study, full-length methanogenesis-related gene sequences were clustered into GSCs at 97% nucleotide identity to represent closely related functional variants in a consistent manner across genes and samples.

This threshold was selected for two reasons. First, 97% identity is a widely used and relatively conservative operational benchmark for grouping highly similar sequences into comparable analytical units [14]. Second, because functional genes often evolve more rapidly than conserved ribosomal markers, very high similarity thresholds may over-split closely related variants. A 97% identity threshold therefore provides a practical balance between retaining phylogenetically informative variation and avoiding excessive fragmentation of highly similar sequences.

**Supplementary Figures**


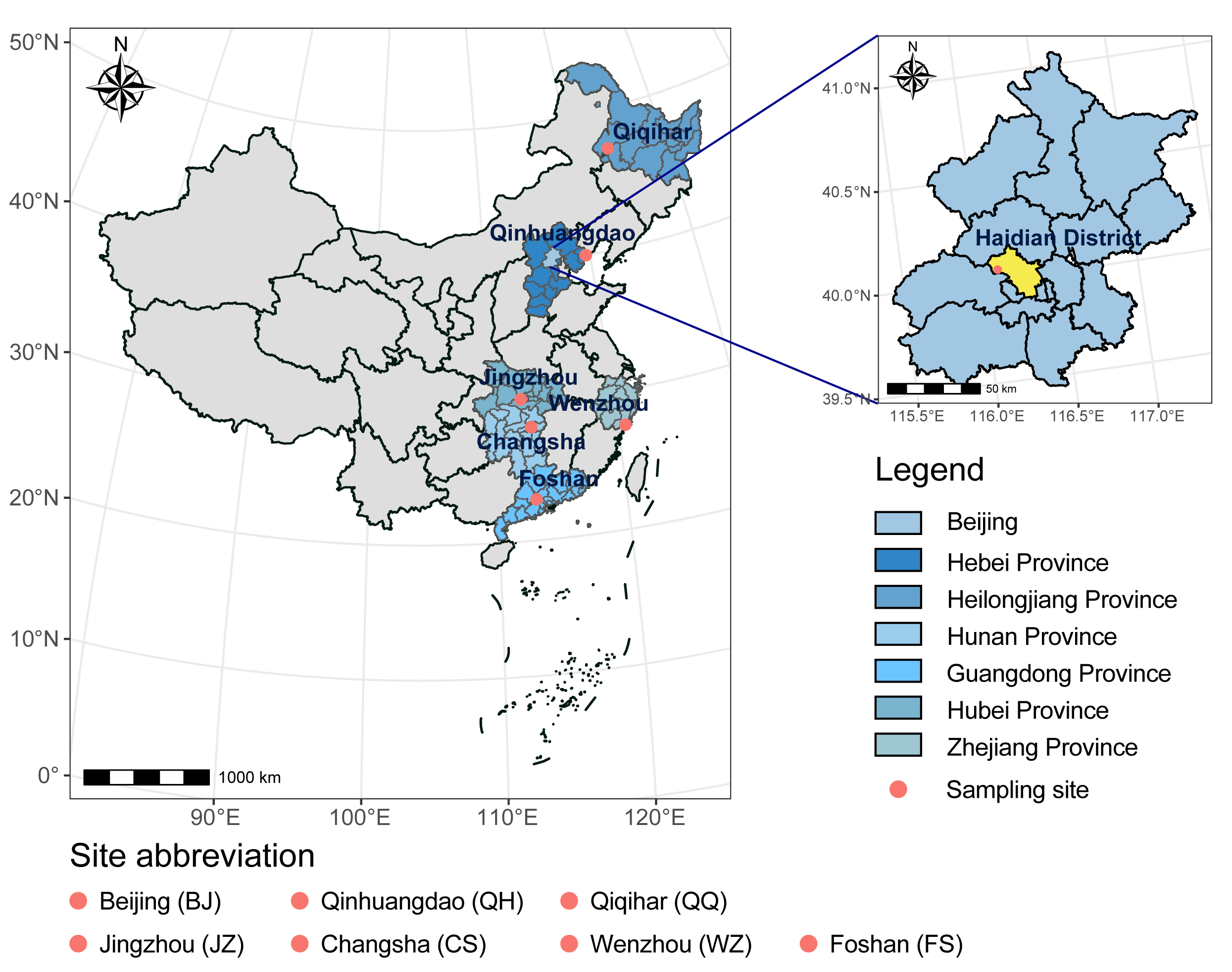


**Figure S1.** Geographical distribution of 7 sites where anaerobic digestion samples were collected.


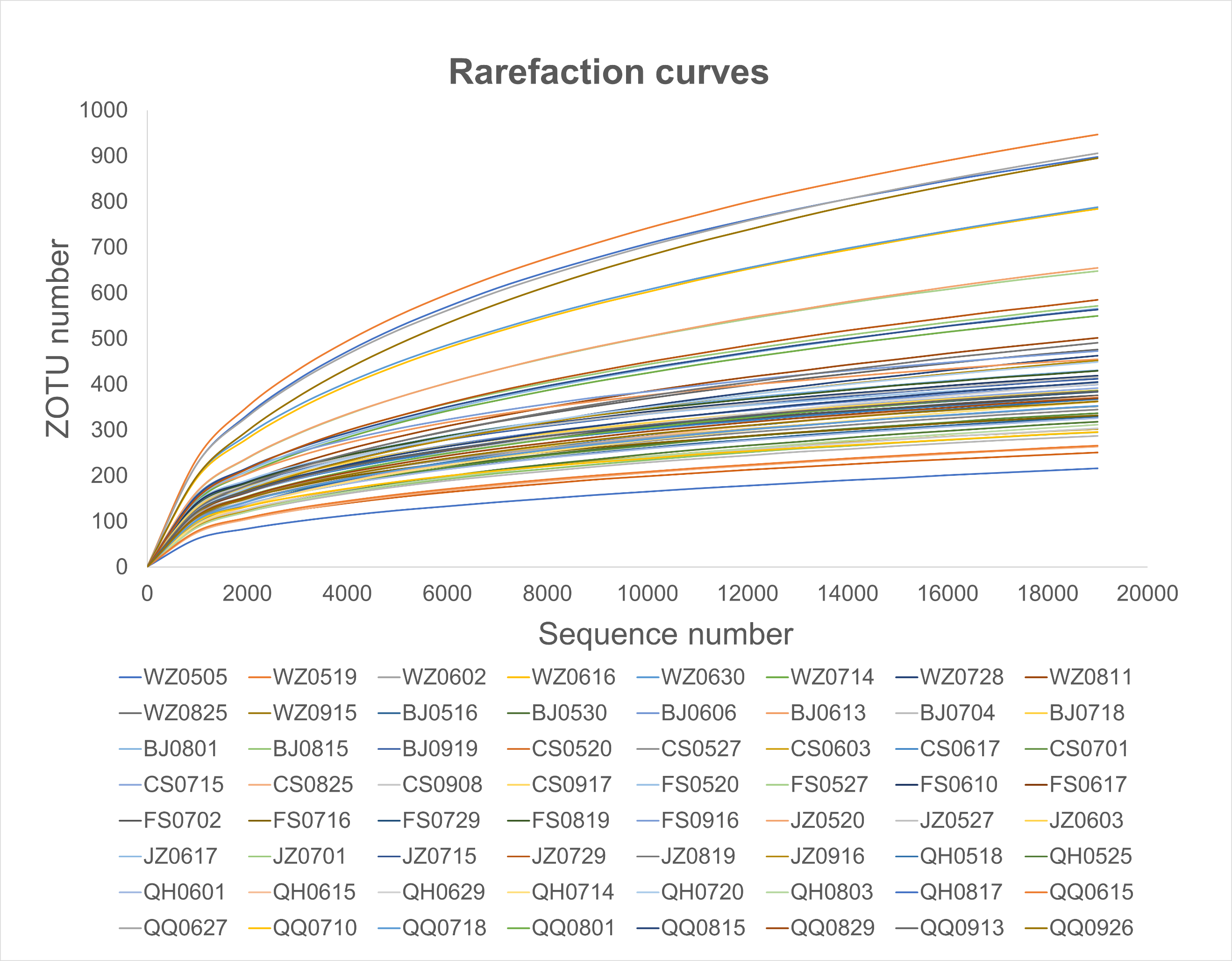


**Figure S2.** Rarefaction curves of 16S rRNA gene amplicon sequences from 64 anaerobic digestion sludge samples.


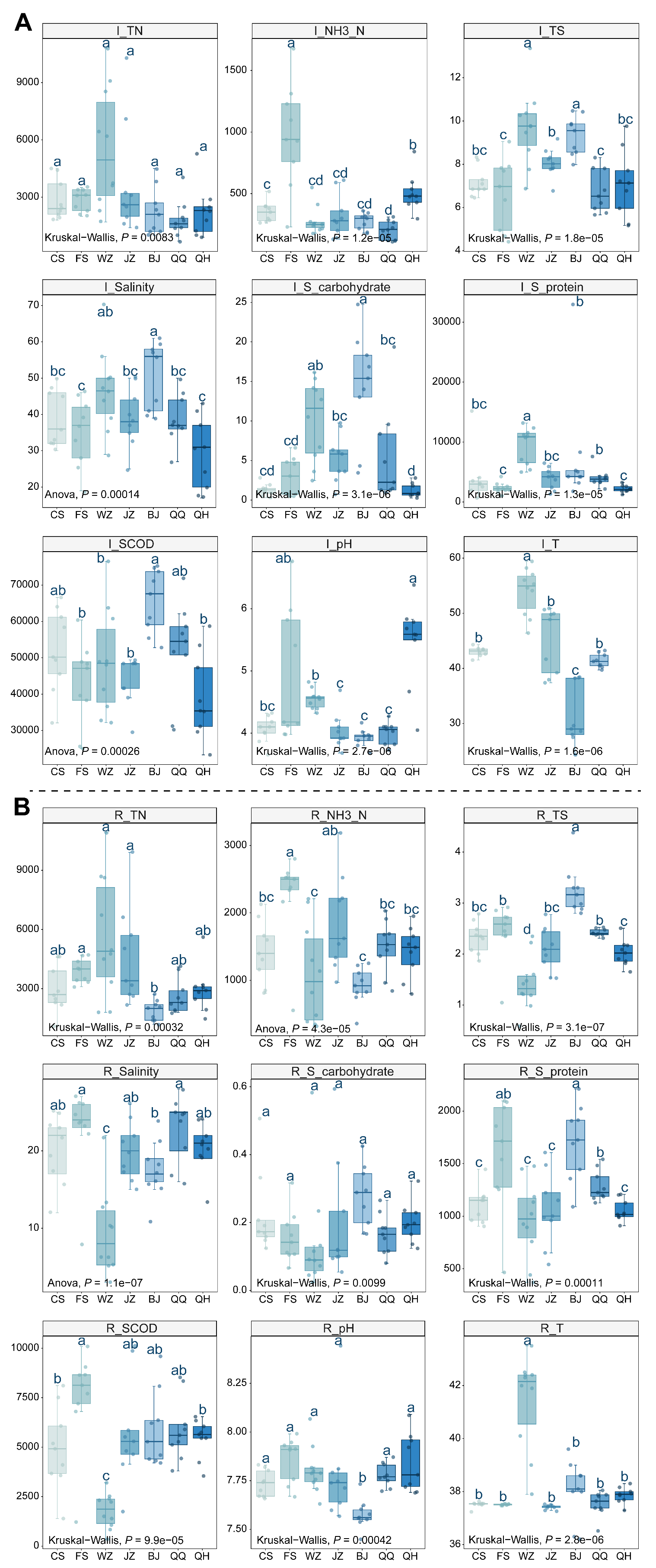


**Figure S3.** Variation in influent (A) and in-reactor (B) characteristics across seven full-scale FW-AD systems. Measured physicochemical parameters include pH, salinity (‰), total nitrogen (TN, mg/L), ammonia nitrogen (NH_3_-N, mg/L), total solids (TS, %), soluble chemical oxygen demand (SCOD, mg/L), soluble carbohydrate (S-carbohydrate, g/L), and soluble protein (S-protein, μg/mL). Variables prefixed with "I" refer to influent properties, whereas those with "R" represent in-reactor conditions. The influent and in-reactor temperature (T, ℃) were recorded by the online monitoring equipment of the facility, among which the influent temperatures at sites FS and QH were unavailable. Statistical differences across sites were assessed using one-way ANOVA and pairwise Student's *t*-tests when data satisfied normality and homoscedasticity assumptions. For non-normally distributed or heteroscedastic variables, Kruskal–Wallis tests and pairwise Wilcoxon rank-sum tests were applied. All *P* values from pairwise comparisons were adjusted using the false discovery rate (FDR) method. Different letters above the boxes indicate statistically significant differences among sites based on post hoc multiple comparisons (*P* < 0.05); sites sharing the same letter are not significantly different.


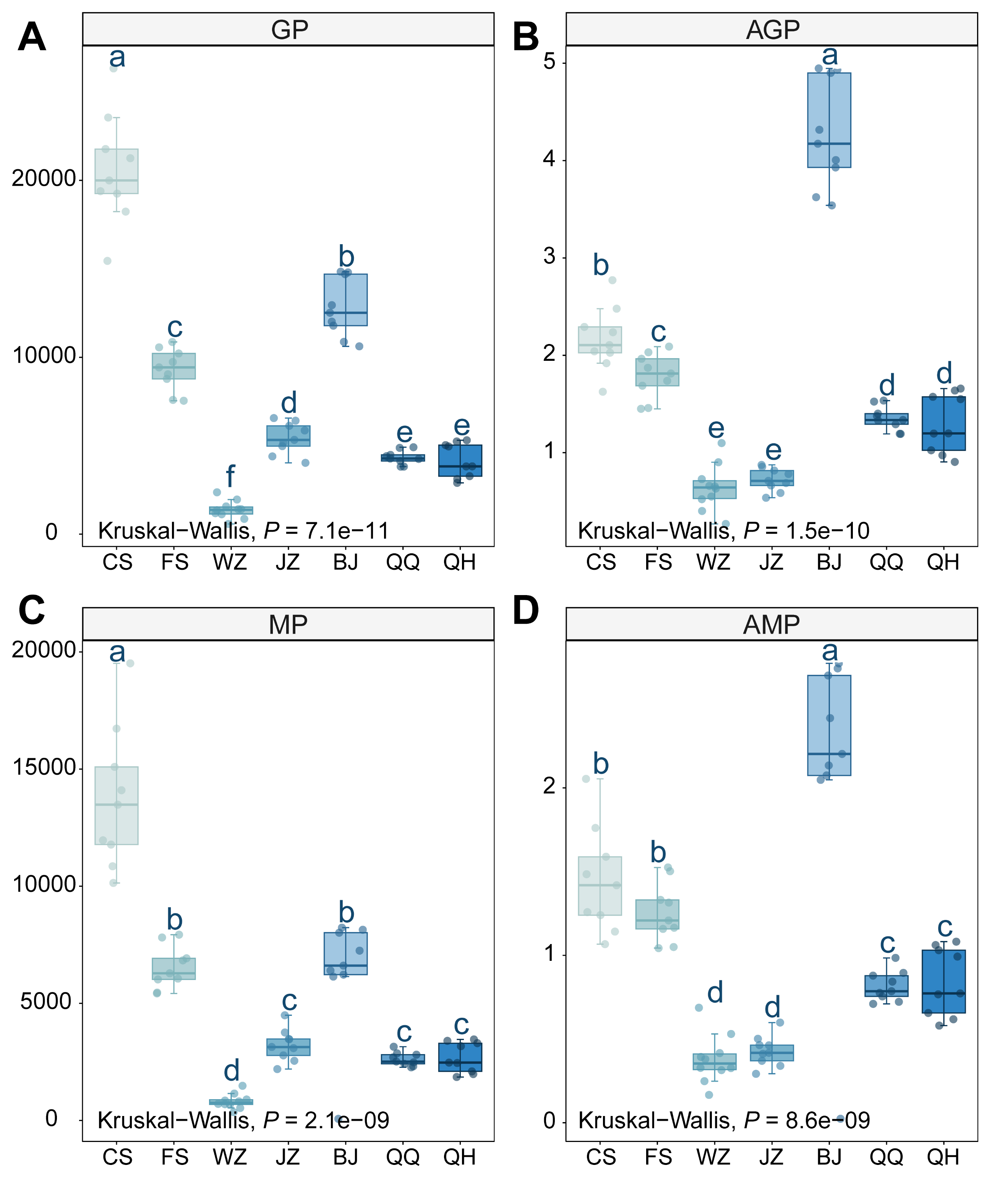


**Figure S4.** Biogas and methane production performance across seven full-scale FW-AD systems. GP: gas production (m^3^/d), AGP: average gas production (m^3^/m^3^•d^-1^), MP: methane production (m^3^/d), AMP: average methane production (m^3^/m^3^•d^-1^). Statistical significance is indicated by different letters as described in Figure S3.


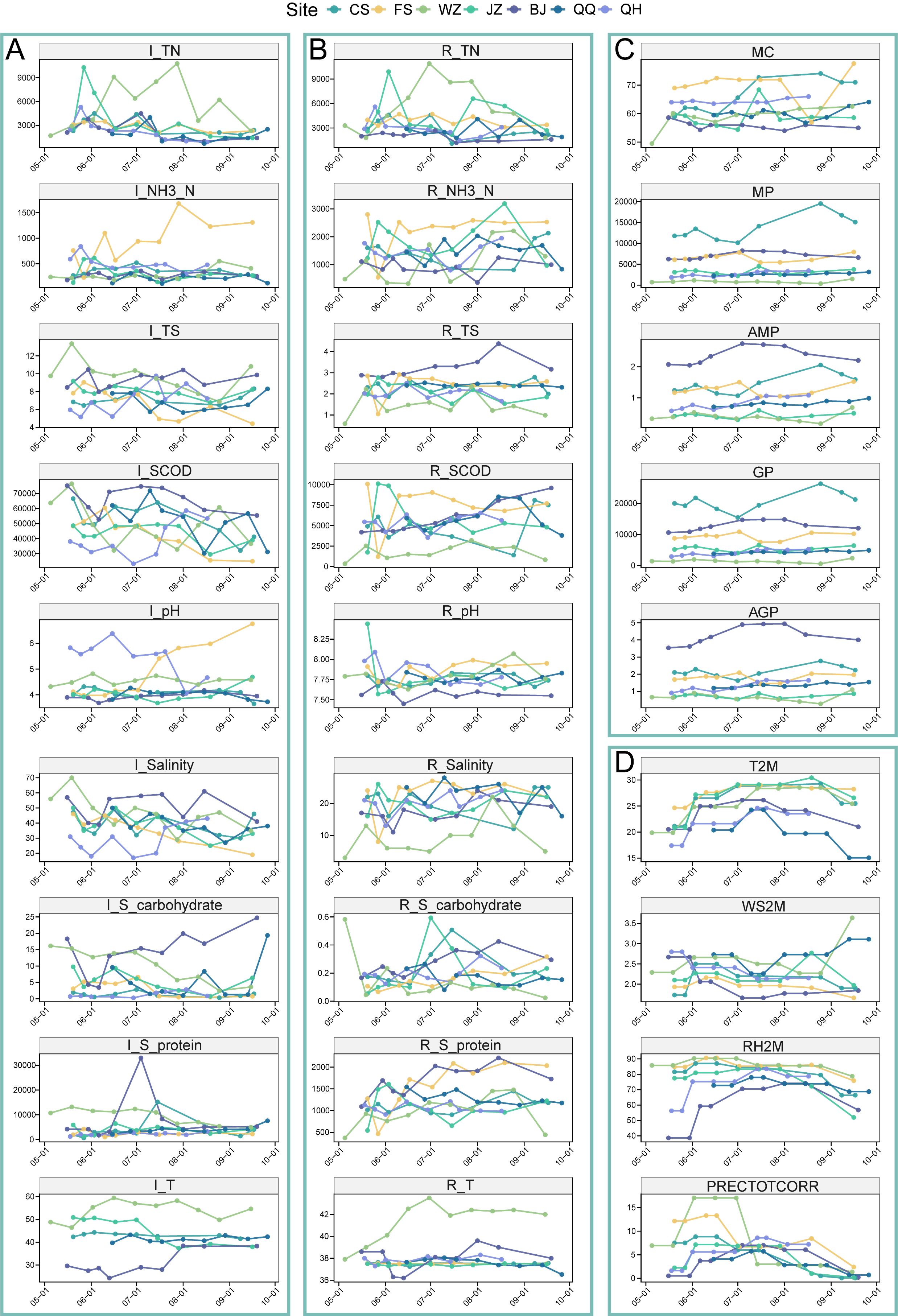


**Figure S5.** Temporal dynamics of environmental parameters and performance indicators across seven full-scale AD systems. Time-series profiles of influent characteristics (A), in-reactor environmental factors (B), gas production performance indicators (C), and climatic variables (D) measured from May to September 2022. Each line represents repeated measurements at one facility during the sampling period. Physicochemical parameters include pH, salinity (‰), total nitrogen (TN, mg/L), ammonia nitrogen (NH_3_-N, mg/L), total solids (TS, %), soluble chemical oxygen demand (SCOD, mg/L), soluble carbohydrate (S-carbohydrate, g/L), soluble protein (S-protein, μg/mL), and temperature (T, ℃). Variables prefixed with "I" refer to influent characteristics, whereas those with "R" represent in-reactor environmental factors. Performance indicators include methane content (MC, %), gas production (GP, m^3^/d), average gas production (AGP, m^3^/m^3^•d^-1^), methane production (MP, m^3^/d), and average methane production (AMP, m^3^/m^3^•d^-1^). Climatic variables include 2-meter monthly mean air temperature (T2M), wind speed (WS2M), relative humidity (RH2M), and corrected precipitation (PRECTOTCORR).


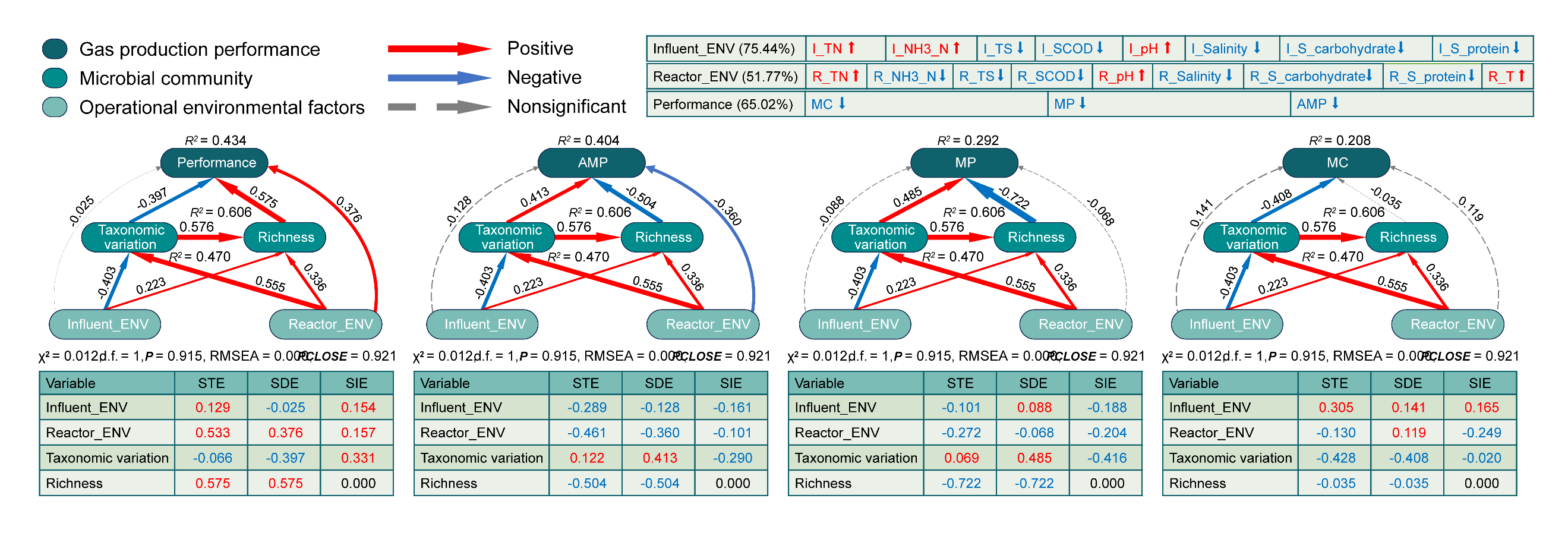


**Figure S6.** Structural equation modeling (SEM) showing the relationships among community composition, environmental variables and gas production performance in anaerobic digestion systems. (A) Overall gas production performance. (B) average methane production (AMP). (C) methane production (MP). (D) methane content (MC). Red and blue arrows indicate significant positive and negative effects (*P* < 0.05), respectively, whereas gray dashed lines represent non-significant paths. The numbers beside the arrows represent standardized path coefficients (*β*), and arrow thickness is proportional to the strength of the effect. *R*^2^ denotes the proportion of variance explained for each endogenous variable. Standardized total effects (STE), standardized direct effects (SDE), standardized indirect effects (SIE), and model fit indices are listed below each SEM panel. Environmental variables are categorized into influent (Influent_ENV) and in-reactor (Reactor_ENV) factors, including total nitrogen (TN), ammonia nitrogen (NH_3_-N), total solids (TS), soluble chemical oxygen demand (SCOD), pH, salinity, soluble carbohydrate (S-carbohydrate), soluble protein (S-protein), and reactor temperature (T), where the variables beginning with "I" represent influent characteristics, whereas those beginning with "R" represent in-reactor characteristics. Due to multicollinearity between different indicators, overall gas production performance is evaluated using three indicators: AMP, MP, and MC. The taxonomic composition of the microbial community is represented by the first principal coordinate (PC1) from the Bray–Curtis dissimilarity-based principal coordinate analysis (PCoA) (Figure 1B). Similarly, both influent/in-reactor environmental factors and overall gas production performance are represented by the PC1 obtained from Bray-Curtis-based PCoA. The boxes at the top of the figure indicate the proportion of variance explained by the PC1, and blue and red arrows within the box indicate the positive and negative correlations, respectively, for the first principal component and components.


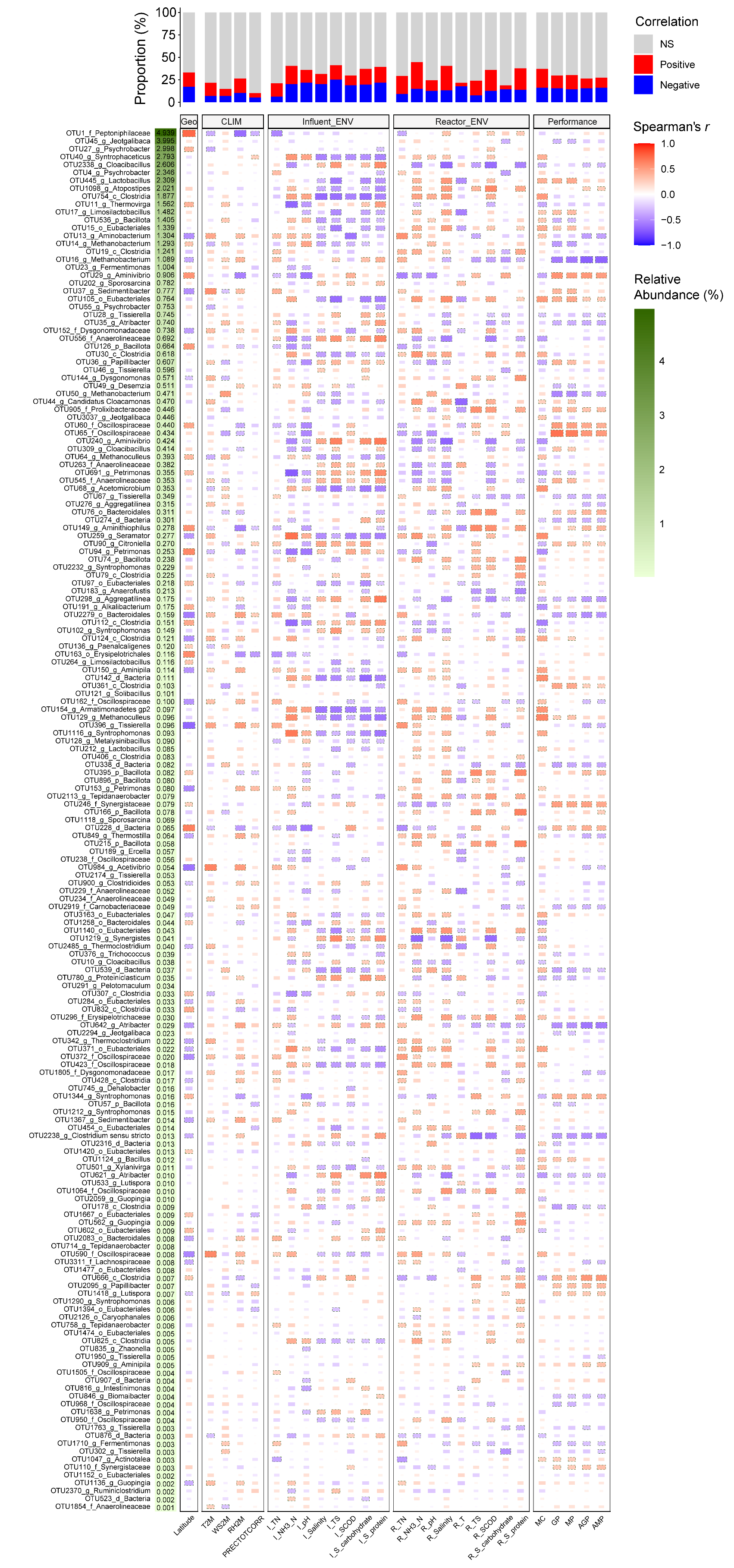


**Figure S7.** Taxon-specific environmental associations of core ZOTUs across AD systems. Spearman correlations between the relative abundances of 175 core ZOTUs (shared by all seven AD systems) and geographic, climatic, and influent/in-reactor environmental variables. Color gradients and box sizes represent correlation coefficients. Statistically significant correlations (*P* < 0.05) are indicated by dashed outlines. Bar plots at the top show the proportion of taxa significantly positively or negatively correlated with each factor. The mean relative abundance of each ZOTU is displayed as green gradient blocks on the left, ordered from highest to lowest. The lowest assigned taxonomic rank is shown following each ZOTU ID, where d, p, c, o, f, and g denote domain, phylum, class, order, family, and genus, respectively.


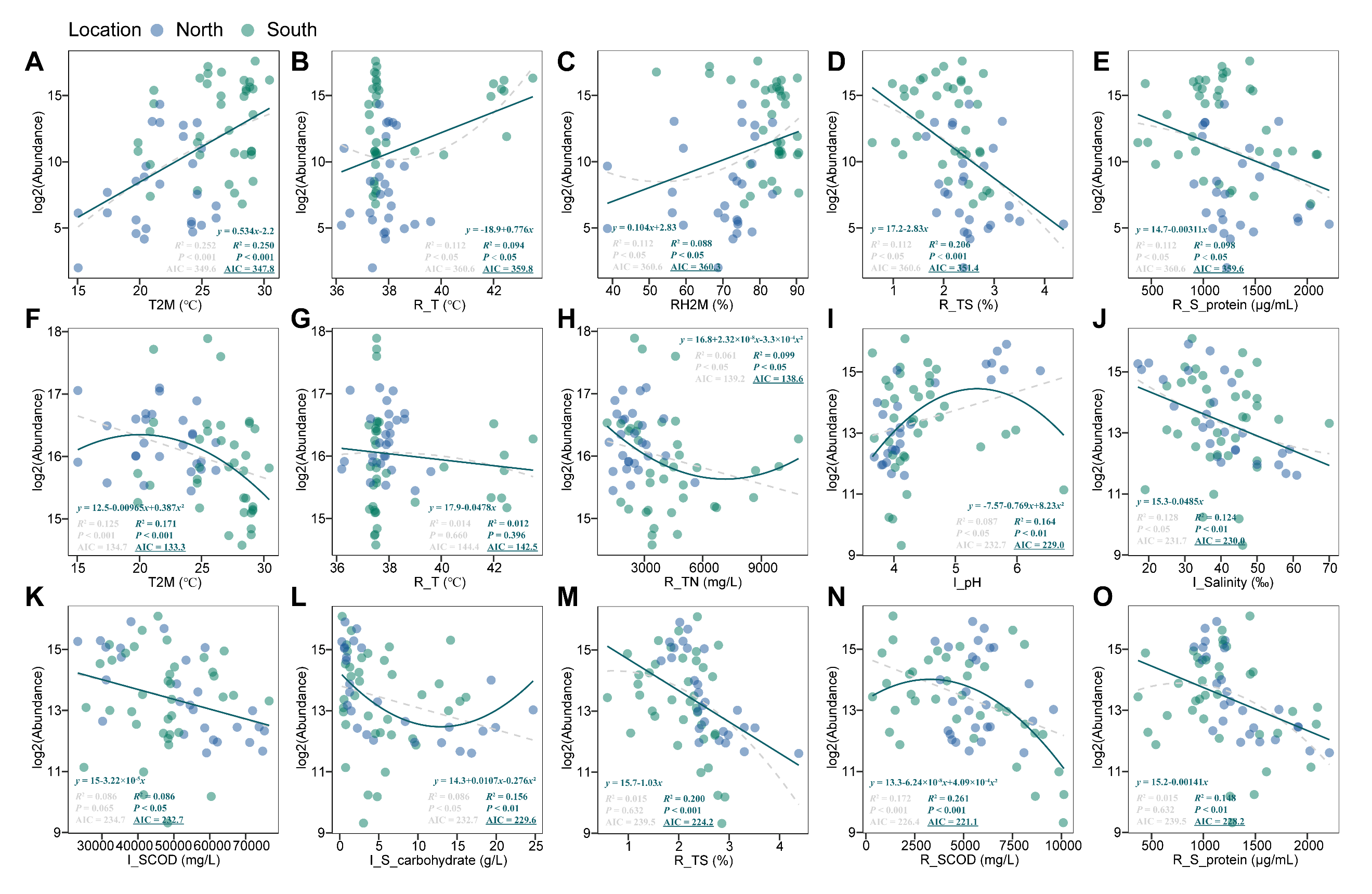


**Figure S8.** Regression analyses between the abundances of different methanogenic pathways and environmental factors. (A-E) Methylotrophic methanogenesis. (F-H) Aceticlastic methanogenesis. (I-O) Hydrogenotrophic methanogenesis. Pathway abundances were log_2_-transformed prior to analysis. Both linear (first-order) and quadratic (second-order) regression models were fitted, with corresponding regression lines, *R*^2^, *P* values, and Akaike Information Criterion (AIC) values indicated in different colors. The best-fit model (based on the lower AIC value) is highlighted in each panel. All samples (n = 64) were categorized into northern (n = 27) and southern (n = 37) sites according to their geographical locations. T2M, monthly average temperature; RH2M, monthly average relative humidity.


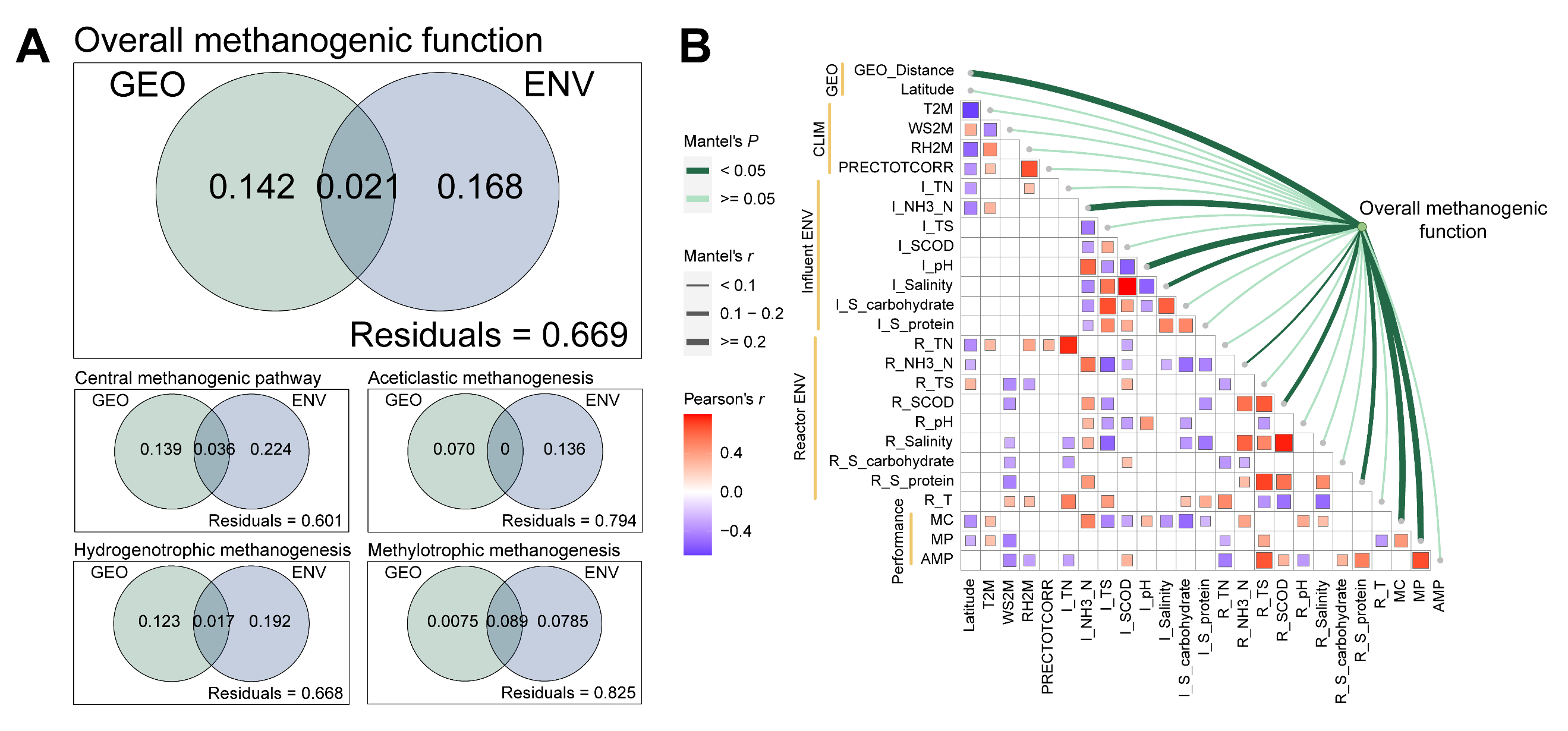


**Figure S9.** Environmental drivers of overall methanogenic functional composition. (A) Variation partitioning analysis (VPA) illustrating the relative contributions of geographic distance and environmental factors to the variation in overall methanogenic functional composition. (B) Partial Mantel tests assessing the associations between the geographic distance-corrected functional composition of the overall methanogenic pathway and individual factors. Climate factors (CLIM) include monthly average temperature (T2M), monthly average wind speed (WS2M), monthly average relative humidity (RH2M), and corrected monthly total precipitation (PRECTOTCORR). Influent/in-reactor physicochemical properties (Influent/Reactor ENV) include total nitrogen (TN), ammonia nitrogen (NH_3_-N), total solids (TS), soluble chemical oxygen demand (SCOD), pH, salinity, soluble carbohydrate (S-carbohydrate), soluble protein (S-protein), and reactor temperature (T). Gas production performance (Performance) includes methane content (MC), methane production (MP), and average methane production (AMP). The thickness of the lines reflects the strength of the correlation. The color of the lines reflects the statistical significance of this relationship. The Spearman's rank correlation coefficients between pairwise factors are expressed as color gradients and only significantly correlated relationships are shown in colored boxes.


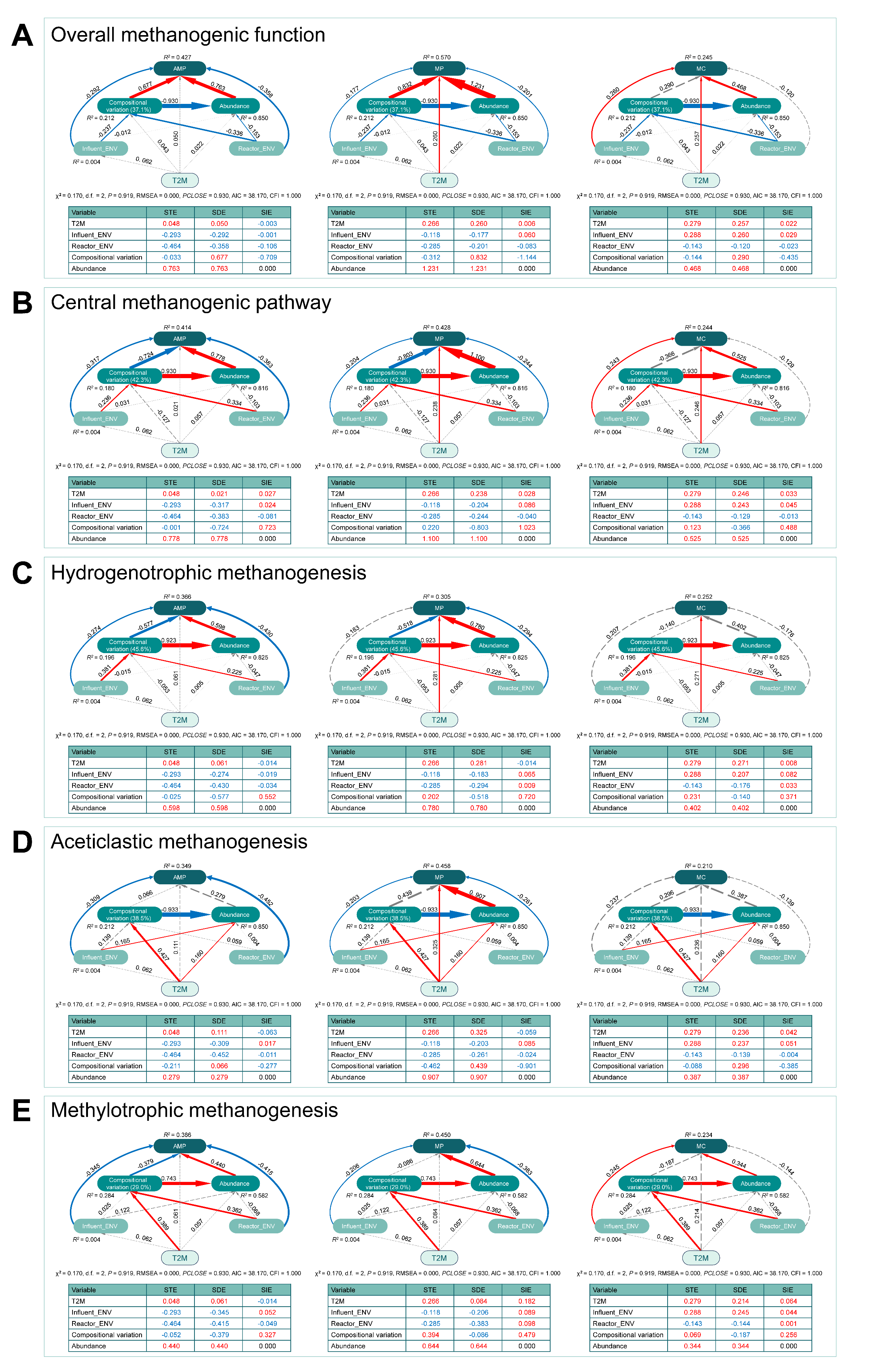


**Figure S10.** SEMs depicting the relationships between functional composition of the overall methanogenic function (A), central methanogenesis (B), hydrogenotrophic methanogenesis (C), aceticlastic methanogenesis (D), and methylotrophic methanogenesis (E), and environmental factors, and three gas production performance indicators. Functional composition is represented by the first principal coordinate (PC1) derived from Bray-Curtis-based PCoA, with the proportion of variance explained by PC1 indicated in parentheses.


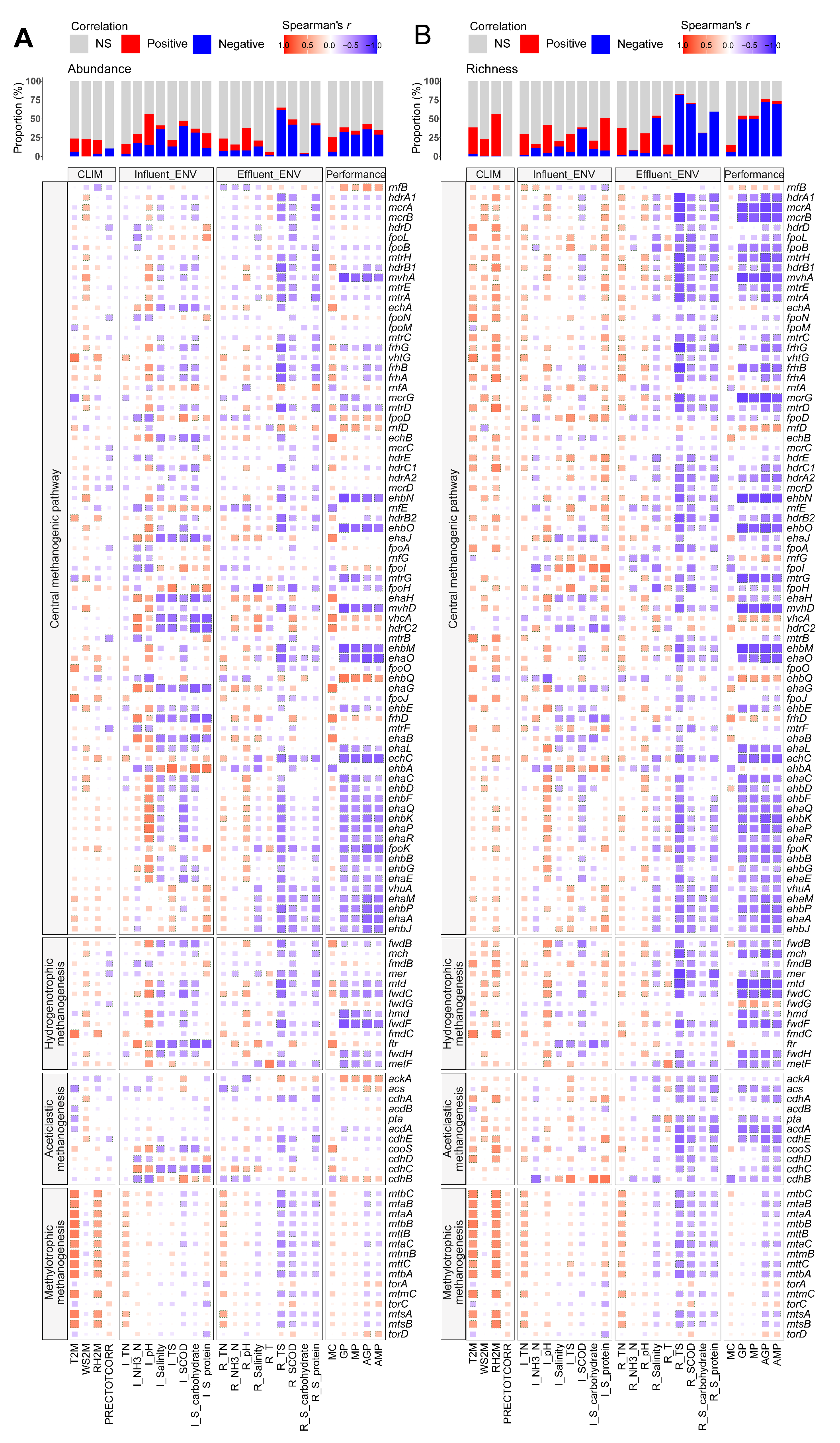


**Figure S11.** Spearman correlations between the abundance (A) and richness (B) of each functional gene and environmental factors, and gas production performance. Correlation coefficients are indicated by a color gradient and box size. Statistically significant correlations (*P* < 0.05) are highlighted with dashed boxes. Bar plots on the left indicate the proportion of functional genes significantly positively or negatively correlated with each factor. *P* values were adjusted using the FDR method.


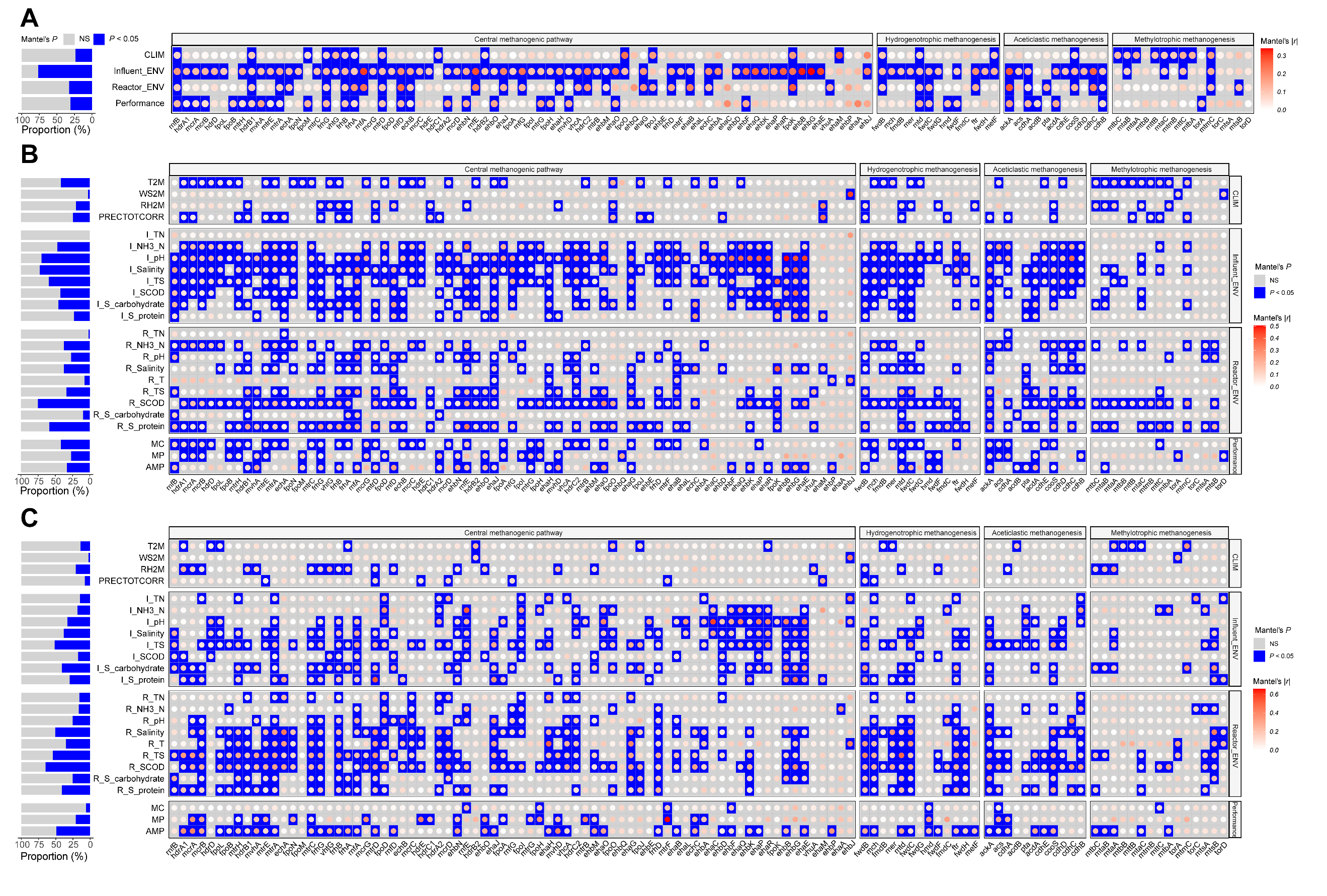


**Figure S12.** Partial Mantel tests between the compositional and phylogenetic variation (geographic distance-corrected) of each functional gene and environmental factors, and gas production performance. (A) Partial Mantel tests between the compositional variation of each functional gene and different categories of factors. (B, C) Partial Mantel tests between functional gene compositional variation (B) and phylogenetic variation (C) and individual factors. Mantel's |*r*| values are visualized using color gradients, with significant correlations highlighted in blue. Bar plots on the left indicate the proportion of genes significantly associated with each factor.


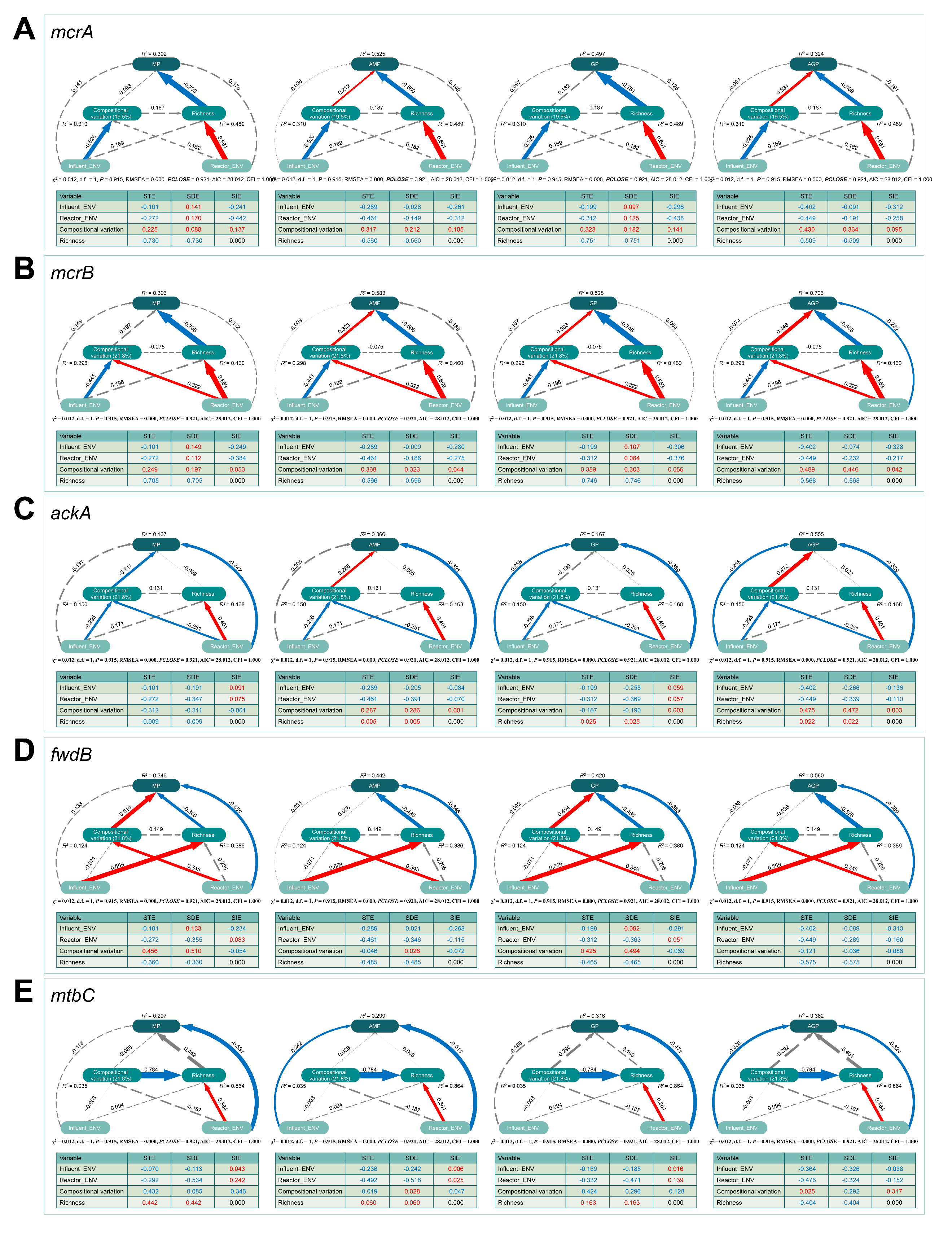


**Figure S13.** SEMs illustrating the relationships between compositional variation of representative genes from different methanogenic pathways, environmental factors, and gas production performance. (A, B) *mcrA* (A) and *mcrB* (B), two key functional genes involved in the central methanogenesis pathway. (C-E) *ackA* (C) from the aceticlastic methanogenesis pathway, *fwdB* (D) from the hydrogenotrophic methanogenesis pathway, and *mtbC* (E) from the methylotrophic methanogenesis pathway, which represent the most abundant functional genes within their respective pathways. Compositional variation of each gene is represented by the PC1 derived from Bray-Curtis-based PCoA, with the proportion of variance explained by PC1 indicated in parentheses.


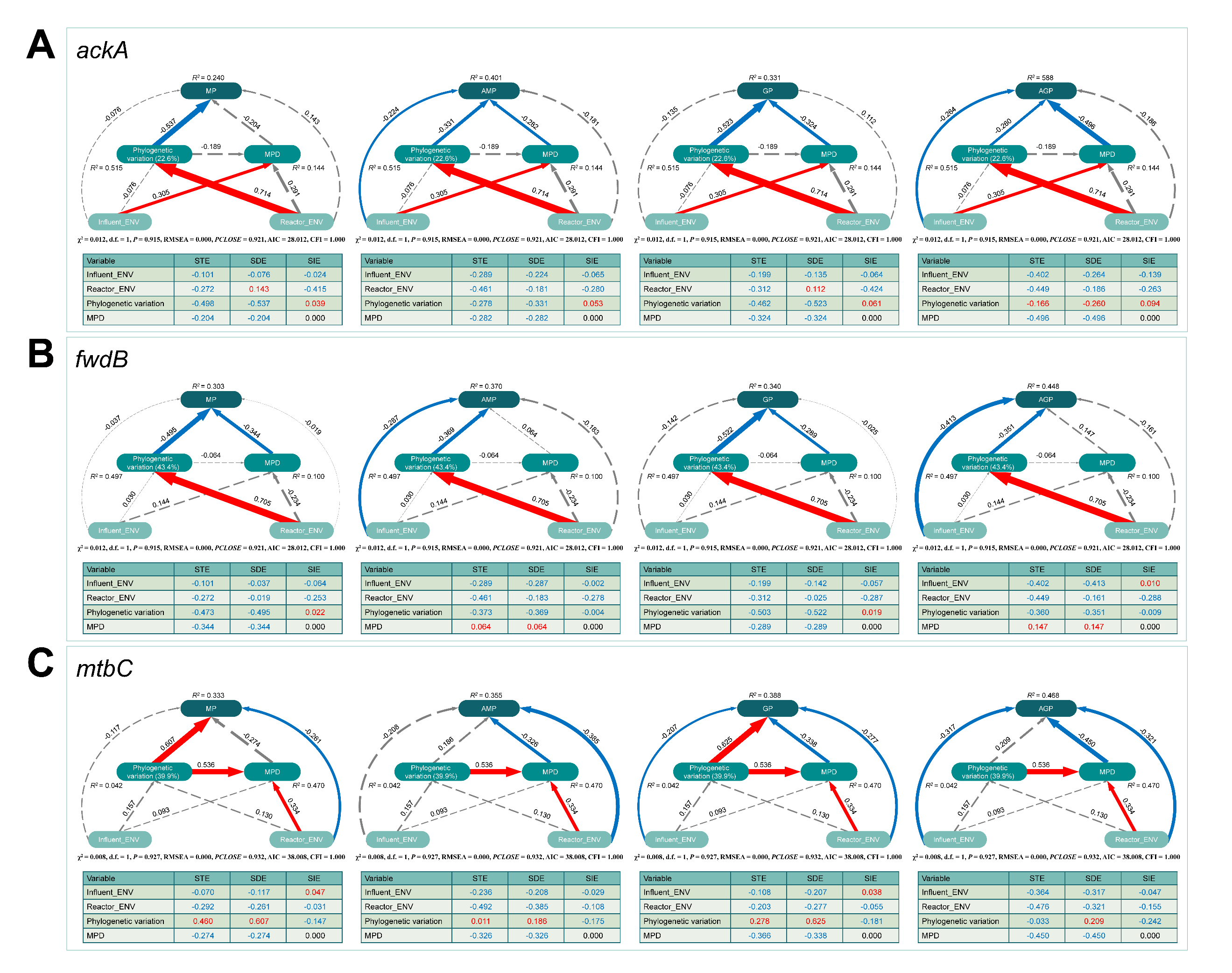


**Figure S14.** SEMs illustrating the relationships between phylogenetic variation of representative genes from different methanogenic pathways, environmental factors, and gas production performance. (A) *ackA* from the aceticlastic methanogenesis pathway, (B) *fwdB* from the hydrogenotrophic methanogenesis pathway, and (C) *mtbC* from the methylotrophic methanogenesis pathway, representing the most abundant functional genes within their respective pathways. Phylogenetic variation is represented by the PC1 derived from unweighted UniFrac-based PCoA, with the proportion of variance explained by PC1 indicated in parentheses.


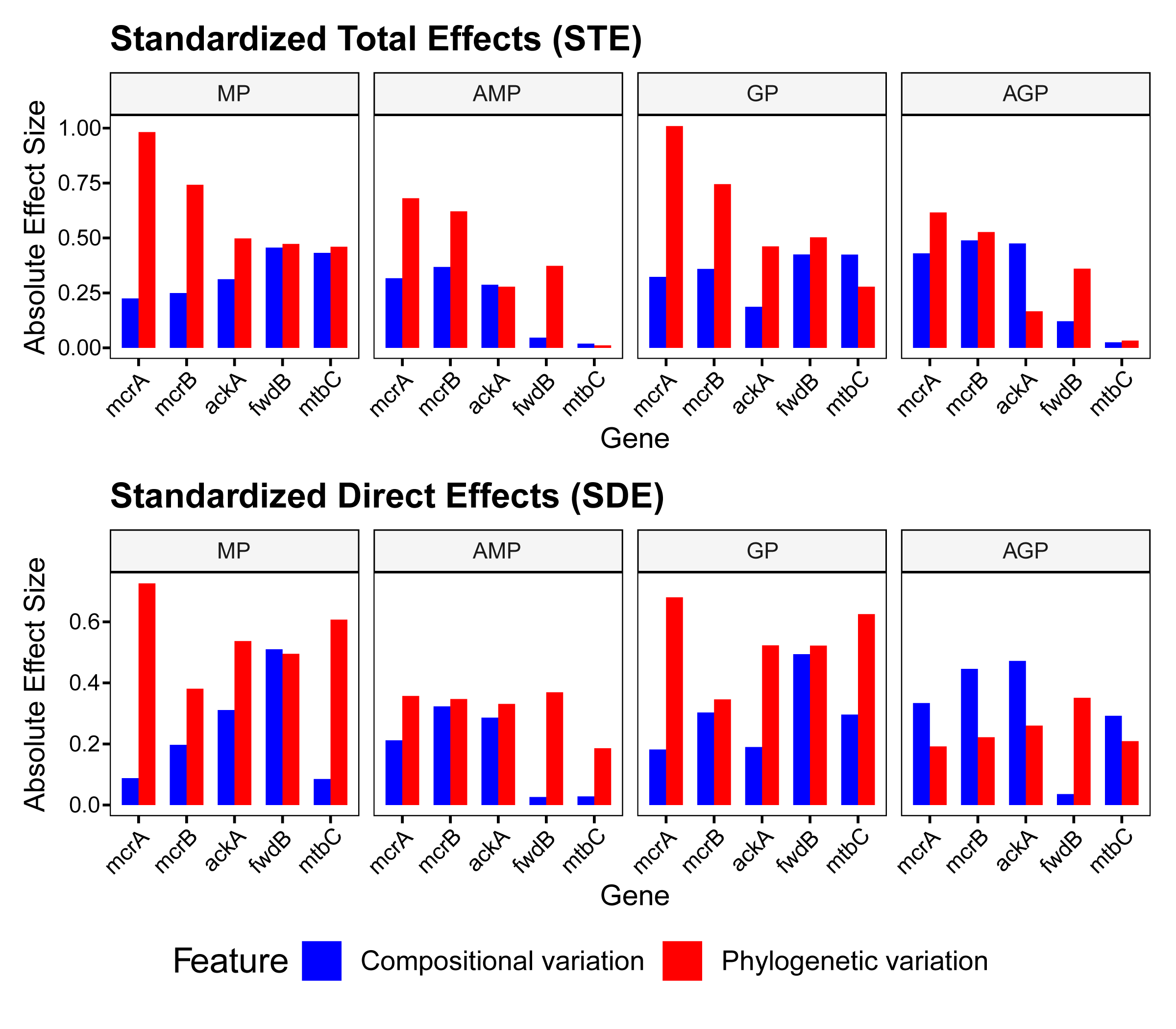


**Figure S15.** Comparison of the standardized total (A) and direct (B) effects of compositional and phylogenetic variations of representative methanogenesis genes on gas production performance.

**Supplementary Tables**

**Table S1** Geographical location and sampling information of seven anaerobic digestion reactors for food waste.

| **Sampling site** | **Site abbreviation** | **Geographical coordinates** | **Sample ID** | **Sampling Date** | **Effective volume (m^3^)** | **Digester samples** | **Influent samples** |
| --- | --- | --- | --- | --- | --- | --- | --- |
| Beijing | BJ | N40°2′58″, E116°6′31″ | BJ0516 | 2022-05-16 | 3000 | 9 | 9 |
|  |  |  | BJ0530 | 2022-05-30 |  |  |  |
|  |  |  | BJ0606 | 2022-06-06 |  |  |  |
|  |  |  | BJ0613 | 2022-06-13 |  |  |  |
|  |  |  | BJ0704 | 2022-07-04 |  |  |  |
|  |  |  | BJ0718 | 2022-07-18 |  |  |  |
|  |  |  | BJ0801 | 2022-08-01 |  |  |  |
|  |  |  | BJ0815 | 2022-08-15 |  |  |  |
|  |  |  | BJ0919 | 2022-09-19 |  |  |  |
| Qinhuangdao | QH | N40°0′17″, E119°37′59″ | QH0518 | 2022-05-18 | 3200 | 9 | 9 |
|  |  |  | QH0525 | 2022-05-25 |  |  |  |
|  |  |  | QH0601 | 2022-06-01 |  |  |  |
|  |  |  | QH0615 | 2022-06-15 |  |  |  |
|  |  |  | QH0629 | 2022-06-29 |  |  |  |
|  |  |  | QH0714 | 2022-07-14 |  |  |  |
|  |  |  | QH0720 | 2022-07-20 |  |  |  |
|  |  |  | QH0803 | 2022-08-03 |  |  |  |
|  |  |  | QH0817 | 2022-08-17 |  |  |  |
| Qiqihar | QQ | N47°10′0″, E124°1′1″ | QQ0615 | 2022-06-15 | 3200 | 9 | 9 |
|  |  |  | QQ0627 | 2022-06-27 |  |  |  |
|  |  |  | QQ0710 | 2022-07-10 |  |  |  |
|  |  |  | QQ0718 | 2022-07-18 |  |  |  |
|  |  |  | QQ0801 | 2022-08-01 |  |  |  |
|  |  |  | QQ0815 | 2022-08-15 |  |  |  |
|  |  |  | QQ0829 | 2022-08-29 |  |  |  |
|  |  |  | QQ0913 | 2022-09-13 |  |  |  |
|  |  |  | QQ0926 | 2022-09-26 |  |  |  |
| Foshan | FS | N22°59′52″, E113°0′6″ | FS0520 | 2022-05-20 | 5200 | 9 | 9 |
|  |  |  | FS0527 | 2022-05-27 |  |  |  |
|  |  |  | FS0610 | 2022-06-10 |  |  |  |
|  |  |  | FS0617 | 2022-06-17 |  |  |  |
|  |  |  | FS0702 | 2022-07-02 |  |  |  |
|  |  |  | FS0716 | 2022-07-16 |  |  |  |
|  |  |  | FS0729 | 2022-07-29 |  |  |  |
|  |  |  | FS0819 | 2022-08-19 |  |  |  |
|  |  |  | FS0916 | 2022-09-16 |  |  |  |
| Wenzhou | WZ | N27°30′44″, E120°37′53″ | WZ0505 | 2022-05-05 | 2154 | 10 | 10 |
|  |  |  | WZ0519 | 2022-05-19 |  |  |  |
|  |  |  | WZ0602 | 2022-06-02 |  |  |  |
|  |  |  | WZ0616 | 2022-06-16 |  |  |  |
|  |  |  | WZ0630 | 2022-06-30 |  |  |  |
|  |  |  | WZ0714 | 2022-07-14 |  |  |  |
|  |  |  | WZ0728 | 2022-07-28 |  |  |  |
|  |  |  | WZ0811 | 2022-08-11 |  |  |  |
|  |  |  | WZ0825 | 2022-08-25 |  |  |  |
|  |  |  | WZ0915 | 2022-09-15 |  |  |  |
| Changsha | CS | N28°15′31″, E113°1′36″ | CS0520 | 2022-05-20 | 9500 | 9 | 9 |
|  |  |  | CS0527 | 2022-05-27 |  |  |  |
|  |  |  | CS0603 | 2022-06-03 |  |  |  |
|  |  |  | CS0617 | 2022-06-17 |  |  |  |
|  |  |  | CS0701 | 2022-07-01 |  |  |  |
|  |  |  | CS0715 | 2022-07-15 |  |  |  |
|  |  |  | CS0825 | 2022-08-25 |  |  |  |
|  |  |  | CS0908 | 2022-09-08 |  |  |  |
|  |  |  | CS0917 | 2022-09-17 |  |  |  |
| Jingzhou | JZ | N30°19′58″, E112°20′48″ | JZ0520 | 2022-05-20 | 7500 | 9 | 9 |
|  |  |  | JZ0527 | 2022-05-27 |  |  |  |
|  |  |  | JZ0603 | 2022-06-03 |  |  |  |
|  |  |  | JZ0617 | 2022-06-17 |  |  |  |
|  |  |  | JZ0701 | 2022-07-01 |  |  |  |
|  |  |  | JZ0715 | 2022-07-15 |  |  |  |
|  |  |  | JZ0729 | 2022-07-29 |  |  |  |
|  |  |  | JZ0819 | 2022-08-19 |  |  |  |
|  |  |  | JZ0916 | 2022-09-16 |  |  |  |

*^a^*The sample ID is named after a combination of site abbreviation and sampling date.

**Table S2** The dissimilarity test of community structure of the seven sites based on permutational multivariate analysis of variances (PERMANOVA).

| **Dissimilarity index** | **Site** | **BJ** | **QH** | **CS** | **FS** | **WZ** | **QQ** | **JZ** |
| --- | --- | --- | --- | --- | --- | --- | --- | --- |
| **Bray-Curtis** | **BJ** | 0.0 | **0.001** | **0.001** | **0.001** | **0.001** | **0.001** | **0.001** |
|  | **QH** | 19.8 | 0.0 | **0.001** | **0.001** | **0.001** | **0.001** | **0.001** |
|  | **CS** | 16.8 | 18.8 | 0.0 | **0.001** | **0.001** | **0.001** | **0.001** |
|  | **FS** | 14.0 | 10.0 | 9.3 | 0.0 | **0.001** | **0.001** | **0.011** |
|  | **WZ** | 13.5 | 14.1 | 11.1 | 7.9 | 0.0 | **0.002** | **0.002** |
|  | **QQ** | 20.9 | 18.6 | 16.0 | 12.4 | 15.5 | 0.0 | **0.001** |
|  | **JZ** | 9.1 | 10.4 | 8.3 | 4.5 | 3.6 | 9.0 | 0.0 |
| **Unweighted UniFrac** | **BJ** | 0.0 | **0.001** | **0.001** | **0.001** | **0.001** | **0.001** | **0.001** |
|  | **QH** | 14.4 | 0.0 | **0.001** | **0.001** | **0.001** | **0.001** | **0.001** |
|  | **CS** | 11.2 | 11.8 | 0.0 | **0.001** | **0.001** | **0.001** | **0.002** |
|  | **FS** | 11.6 | 7.8 | 8.9 | 0.0 | **0.001** | **0.001** | **0.012** |
|  | **WZ** | 13.5 | 12.3 | 13.5 | 9.2 | 0.0 | **0.001** | **0.001** |
|  | **QQ** | 12.9 | 11.9 | 9.7 | 6.8 | 12.5 | 0.0 | **0.001** |
|  | **JZ** | 8.6 | 8.1 | 7.5 | 4.5 | 3.9 | 7.0 | 0.0 |

*^a^*The values of upper triangular matrices are *P* values. The values of lower triangular matrices are *F* values for PERMANOVA. Items with significant differences (*P* < 0.05) are highlighted in bold.

**Table S3** Partial Mantel tests of taxonomic dissimilarity and phylogenetic dissimilarity with different categories of factors.

| **Dissimilarity index** | **Category of factors** | **Mantel's *r*** | ***P* value** |
| --- | --- | --- | --- |
| **Bray-Curtis** | **Geo_Distance** | 0.663 | 0.001 |
|  | **CLIM** | 0.043 | 0.08 |
|  | **Influent_ENV** | 0.299 | 0.001 |
|  | **Reactor_ENV** | 0.267 | 0.001 |
|  | **Performance** | 0.158 | 0.001 |
| **Unweighted UniFrac** | **Geo_Distance** | 0.445 | 0.001 |
|  | **CLIM** | 0.070 | 0.127 |
|  | **Influent_ENV** | 0.276 | 0.001 |
|  | **Reactor_ENV** | 0.542 | 0.001 |
|  | **Performance** | 0.026 | 0.281 |

*^a^*All factors are divided into five categories: geographical distance (Geo_Distance), climatic factors (CLIM), influent characteristics (Influent_ENV), in-reactor characteristics (Reactor_ENV), and gas production performance (Performance). CLIM includes monthly average temperature (T2M), monthly average wind speed (WS2M), monthly average relative humidity (RH2M), and corrected monthly total precipitation (PRECTOTCORR). Influent_ENV/Reactor_ENV includes total nitrogen (TN), ammonia nitrogen (NH_3_-N), total solids (TS), soluble chemical oxygen demand (SCOD), pH, salinity, soluble carbohydrate (S-carbohydrate), soluble protein (S-protein), and reactor temperature (T). Performance includes methane content (MC), methane production (MP), and average methane production (AMP).

*^b^*The categories of factors significantly associated with community dissimilarity (*P* < 0.05) are highlighted in red.

**Table S4** Results of the multiple regression on matrices (MRM) analysis between community dissimilarity and abiotic factors.

| **Category** | **Factors** | **Taxonomic and phylogenetic dissimilarities** | |
| --- | --- | --- | --- |
|  |  | **Bray-Curtis** | **Unweighted UniFrac** |
|  |  | ***R*^2^=0.61***** | ***R*^2^=0.62***** |
| **Geo_Distance** | **Geo_Distance** | 0.044*** | 0.022*** |
| **Influent_ENV** | **I_TN** | 0.008* | NS |
|  | **I_TS** | 0.010** | 0.024*** |
|  | **I_pH** | 0.018*** | NS |
|  | **I_Salinity** | 0.017*** | 0.015* |
| **Reactor_ENV** | **R_TS** | 0.014*** | 0.015** |
|  | **R_SCOD** | 0.017*** | 0.012* |
|  | **R_pH** | NS | 0.012* |
|  | **R_Salinity** | 0.008* | 0.025*** |
|  | **R_S_carbohydrate** | 0.009** | 0.012* |
|  | **R_S_protein** | NS | 0.011* |
|  | **R_T** | -0.009* | NS |
| **CLIM** | **WS2M** | -0.012*** | -0.013** |
|  | **RH2M** | 0.008* | NS |

*^a^*For the abiotic factors, variables beginning with "I" represent influent characteristics, whereas those beginning with "R" represent in-reactor characteristics. WS2M denotes the monthly average wind speed at the sampling site, and RH2M represents the monthly average relative humidity.

*^b^*The overall explanatory power of the abiotic factor matrixes for taxonomic and phylogenetic dissimilarities is reported using *R*^2^ and *P* values.

*^c^*The partial regression coefficients for each factor and their corresponding *P* values are provided.

*^d^*NS: *P* ≥ 0.05, *: *P* < 0.05, **: *P* < 0.01, ***: *P* < 0.001. Only factors with *P* < 0.05 are displayed. Factors significantly associated with both taxonomic and phylogenetic dissimilarities are highlighted in red.

*^e^*Prior to MRM analysis, multicollinearity among explanatory variables was assessed using variance inflation factors (VIFs), with the highest VIF observed for I_Salinity (6.69), indicating an acceptable level of multicollinearity.

**Table S5** The dissimilarity test of functional composition in methanogenic pathways across the seven sites using permutational multivariate analysis of variances (PERMANOVA).

| **Methanogenic pathway** | **Site** | **BJ** | **QH** | **CS** | **FS** | **WZ** | **QQ** | **JZ** |
| --- | --- | --- | --- | --- | --- | --- | --- | --- |
| **Overall methanogenic function** | **BJ** | 0.0 | **0.001** | **0.001** | **0.001** | **0.001** | **0.001** | **0.001** |
|  | **QH** | 41.5 | 0.0 | **0.001** | **0.001** | **0.001** | **0.001** | **0.001** |
|  | **CS** | 16.9 | 14.9 | 0.0 | **0.001** | **0.001** | **0.001** | **0.003** |
|  | **FS** | 11.8 | 27.3 | 18.6 | 0.0 | **0.001** | **0.001** | **0.016** |
|  | **WZ** | 17.1 | 13.9 | 5.7 | 15.5 | 0.0 | **0.001** | **0.047** |
|  | **QQ** | 9.1 | 28.5 | 12.8 | 11.0 | 8.1 | 0.0 | **0.021** |
|  | **JZ** | 4.6 | 10.2 | 5.7 | 3.2 | 2.7 | 2.8 | 0.0 |
| **Central methanogenic pathway** | **BJ** | 0.0 | **0.001** | **0.001** | **0.001** | **0.001** | **0.001** | **0.001** |
|  | **QH** | 89.9 | 0.0 | **0.001** | **0.001** | **0.001** | **0.001** | **0.001** |
|  | **CS** | 24.2 | 17.7 | 0.0 | **0.001** | **0.004** | **0.001** | **0.002** |
|  | **FS** | 18.3 | 35.7 | 21.5 | 0.0 | **0.001** | **0.001** | **0.029** |
|  | **WZ** | 28.5 | 17.4 | 5.5 | 20.4 | 0.0 | **0.001** | **0.019** |
|  | **QQ** | 18.5 | 42.2 | 13.8 | 13.8 | 8.7 | 0.0 | **0.026** |
|  | **JZ** | 6.9 | 12.6 | 6.5 | 3.2 | 3.7 | 2.8 | 0.0 |
| **Aceticlastic methanogenesis** | **BJ** | 0.0 | **0.001** | **0.001** | **0.001** | **0.002** | **0.015** | **0.038** |
|  | **QH** | 14.4 | 0.0 | **0.004** | **0.001** | **0.001** | **0.001** | **0.001** |
|  | **CS** | 7.5 | 7.0 | 0.0 | **0.001** | **0.001** | **0.003** | **0.002** |
|  | **FS** | 10.0 | 14.9 | 14.9 | 0.0 | **0.001** | **0.001** | 0.058 |
|  | **WZ** | 10.1 | 8.6 | 8.0 | 9.6 | 0.0 | **0.001** | 0.167 |
|  | **QQ** | 4.2 | 10.2 | 6.1 | 10.6 | 6.1 | 0.0 | **0.019** |
|  | **JZ** | 3.0 | 6.3 | 6.6 | 2.4 | 1.7 | 3.7 | 0.0 |
| **Hydrogenotrophic methanogenesis** | **BJ** | 0.0 | **0.001** | **0.001** | **0.001** | **0.001** | **0.001** | **0.001** |
|  | **QH** | 140.3 | 0.0 | **0.001** | **0.001** | **0.001** | **0.001** | **0.001** |
|  | **CS** | 32.9 | 27.7 | 0.0 | **0.001** | **0.005** | **0.001** | **0.004** |
|  | **FS** | 16.0 | 26.8 | 15.6 | 0.0 | **0.001** | **0.001** | 0.124 |
|  | **WZ** | 30.7 | 25.9 | 6.8 | 11.8 | 0.0 | **0.002** | **0.023** |
|  | **QQ** | 12.4 | 56.3 | 12.3 | 7.2 | 7.9 | 0.0 | 0.052 |
|  | **JZ** | 7.5 | 15.8 | 7.1 | 1.9 | 4.0 | 2.7 | 0.0 |
| **Methylotrophic methanogenesis** | **BJ** | 0.0 | **0.034** | **0.001** | **0.005** | **0.001** | 0.239 | **0.004** |
|  | **QH** | 2.8 | 0.0 | **0.001** | **0.014** | **0.001** | **0.005** | **0.03** |
|  | **CS** | 11.7 | 11.1 | 0.0 | **0.001** | 0.103 | **0.001** | **0.023** |
|  | **FS** | 4.6 | 4.0 | 14.0 | 0.0 | **0.005** | **0.002** | 0.072 |
|  | **WZ** | 8.6 | 8.2 | 2.3 | 6.2 | 0.0 | **0.001** | 0.168 |
|  | **QQ** | 1.3 | 3.4 | 10.4 | 4.8 | 7.8 | 0.0 | **0.001** |
|  | **JZ** | 3.7 | 3.0 | 3.6 | 2.2 | 1.6 | 4.0 | 0.0 |

*^a^*The values of upper triangular matrices are *P* values. The values of lower triangular matrices are *F* value for PERMANOVA.

*^b^*Items with significant differences (*P* < 0.05) are highlighted in bold. Items with no significant differences (*P* ≥ 0.05) are highlighted in red.

**Table S6** Statistical comparisons of phylogenetic features of methanogenesis-related functional genes between AD systems with different gas production performance levels.

| **Category*^a^*** | **Site** | **Performance indicators** | | | | | | | | **Phylogenetic features** | | | |
| --- | --- | --- | --- | --- | --- | --- | --- | --- | --- | --- | --- | --- | --- |
|  |  | **AMP**  **(m^3^/m^3^•d^-1^)** | | **MP**  **(m^3^/d)** | | **AGP**  **(m^3^/m^3^•d^-1^)** | | **GP**  **(m^3^/d)** | | **PD** | | **MPD** | |
| **High-performing systems** | **BJ** | 2.11 ± 0.28 | 1.61 ± 0.12 | 6342.54 ± 832.42 | 8868.62 ± 800.00 | 4.26 ± 0.18 | 2.74 ± 0.23 | 12789.33 ± 552.46 | 14226.63 ± 1007.51 | 1.73 ± 0.25 | 1.82 ± 0.24 | 0.22 ± 0.02 | 0.25 ± 0.02 |
|  | **CS** | 1.45 ± 0.11 |  | 13736.26 ± 1004.21 |  | 2.17 ± 0.11 |  | 20583.22 ± 1048.31 |  | 1.88 ± 0.24 |  | 0.24 ± 0.02 |  |
|  | **FS** | 1.26 ± 0.06 |  | 6527.06 ± 306.53 |  | 1.79 ± 0.08 |  | 9307.33 ± 398.52 |  | 1.89 ± 0.23 |  | 0.31 ± 0.02 |  |
| **Low-performing systems** | **QQ** | 0.82 ± 0.03 | 0.61 ± 0.04 | 2613.41 ± 96.63 | 2292.45 ± 174.29 | 1.35 ± 0.04 | 1.00 ± 0.06 | 4332.61 ± 131.41 | 3762.68 ± 278.75 | 1.66 ± 0.22 | 2.30 ± 0.26 | 0.23 ± 0.02 | 0.30 ± 0.02 |
|  | **QH** | 0.84 ± 0.07 |  | 2688.25 ± 215.85 |  | 1.3 ± 0.1 |  | 4168.44 ± 324.21 |  | 2.23 ± 0.29 |  | 0.29 ± 0.03 |  |
|  | **JZ** | 0.43 ± 0.03 |  | 3215.44 ± 228.48 |  | 0.72 ± 0.04 |  | 5431.18 ± 294.25 |  | 2.41 ± 0.27 |  | 0.31 ± 0.02 |  |
|  | **WZ** | 0.38 ± 0.05 |  | 816.67 ± 99.73 |  | 0.64 ± 0.07 |  | 1382.9 ± 161.82 |  | 2.86 ± 0.30 |  | 0.36 ± 0.03 |  |
| **High- vs. low-performing systems*^b^*** | | *t*(32.29) = 7.78,  *P* < 0.01 | | *t*(28.48) = 8.03,  *P* < 0.01 | | *t*(30.17) = 7.44,  *P* < 0.01 | | *t*(30.01) = 10.01,  *P* < 0.01 | | *t*(122) = -6.97,  *P* < 0.01 | | *t*(122) = -5.15,  *P* < 0.01 | |
|  |  | *W* = 957, *P* < 0.01 | | *W* = 962, *P* < 0.01 | | *W* = 985, *P* < 0.01 | | *W* = 999, *P* < 0.01 | | *V* = 763, *P* < 0.01 | | *V* = 1773, *P* < 0.01 | |

*^a^*Based on Supplemental Figure S4, the seven AD systems were stratified into two categories: three systems with significantly higher methane production performance were designated as high-performing, whereas the remaining four were classified as low-performing.

*^b^*Between-group comparisons were conducted using Student's *t*-test or Wilcoxon rank-sum test, with Welch's *t*-test applied in cases of unequal variances. For phylogenetic features of methanogenesis-related functional genes, paired *t*-tests and Wilcoxon signed-rank tests were performed. Reported statistics include the *t* statistic for *t*-tests, the *W* statistic for Wilcoxon rank-sum tests, and the *V* statistic for Wilcoxon signed-rank tests, along with their corresponding *P* values. Statistically significant differences (*P* < 0.05) are highlighted in red.

*^c^*All performance indicators and phylogenetic metrics are presented as mean ± s.e.m.

**Table S7** Statistical comparison of standardized effect sizes of phylogenetic and compositional variations of representative methanogenesis genes on gas production performance.

| **Effect type** | **Comparison pairs** | **Mean absolute** **standardized effect size** | | ***W* statistic** | ***P* value** |
| --- | --- | --- | --- | --- | --- |
|  |  | **Phylogenetic variation** | **Compositional variation** |  |  |
| **STE** | 20 | 0.491 | 0.298 | 184 | *P* < 0.01 |
| **SDE** | 20 | 0.413 | 0.256 | 173 | *P* < 0.05 |

*^a^*Differences between the absolute standardized effect sizes derived from phylogenetic and compositional variations on gas production performance were assessed using paired Wilcoxon signed-rank tests across 20 gene-indicator pairs. Mean absolute effect sizes, Wilcoxon *W* statistics, and corresponding *P* values are reported. Statistically significant differences (*P* < 0.05) are highlighted in red.

**Table S8** Spearman correlation analysis of α-diversity of prokaryotic community with environmental factors and gas production performance in anaerobic digestion system.

| **Factors** | **α-diversity index** | | | | | | |
| --- | --- | --- | --- | --- | --- | --- | --- |
|  | **Shannon** | **Inv Simpson** | **Richness** | **Pielou evenness** | **Simpson evenness** | **PD** | **MPD** |
| **T2M** | 0.174 (NS) | 0.115 (NS) | 0.215 (NS) | 0.142 (NS) | 0.049 (NS) | 0.01 (NS) | -0.047 (NS) |
| **WS2M** | 0.068 (NS) | 0.187 (NS) | 0.096 (NS) | 0.026 (NS) | 0.114 (NS) | 0.199 (NS) | 0.373 (*) |
| **RH2M** | 0.388 (*) | 0.37 (*) | 0.361 (*) | 0.349 (*) | 0.158 (NS) | 0.16 (NS) | 0.082 (NS) |
| **PRECTOTCORR** | 0.177 (NS) | 0.156 (NS) | 0.103 (NS) | 0.194 (NS) | 0.046 (NS) | 0.047 (NS) | -0.204 (NS) |
| **I_TN** | 0.301 (*) | 0.352 (*) | 0.193 (NS) | 0.304 (*) | 0.298 (NS) | 0.074 (NS) | 0.045 (NS) |
| **I_NH3_N** | -0.145 (NS) | -0.142 (NS) | -0.166 (NS) | -0.045 (NS) | 0.002 (NS) | -0.313 (*) | -0.344 (*) |
| **I_TS** | 0.397 (*) | 0.343 (*) | 0.38 (*) | 0.31 (*) | 0.063 (NS) | 0.427 (**) | 0.151 (NS) |
| **I_SCOD** | -0.065 (NS) | -0.056 (NS) | -0.169 (NS) | -0.029 (NS) | 0.022 (NS) | -0.109 (NS) | -0.111 (NS) |
| **I_pH** | -0.074 (NS) | -0.084 (NS) | 0.088 (NS) | -0.116 (NS) | -0.238 (NS) | 0.023 (NS) | -0.092 (NS) |
| **I_Salinity** | 0.235 (NS) | 0.207 (NS) | 0.139 (NS) | 0.219 (NS) | 0.091 (NS) | 0.132 (NS) | -0.001 (NS) |
| **I_S_carbohydrate** | 0.41 (**) | 0.373 (*) | 0.357 (*) | 0.365 (*) | 0.198 (NS) | 0.38 (*) | 0.105 (NS) |
| **I_S_protein** | 0.362 (*) | 0.306 (*) | 0.447 (**) | 0.217 (NS) | -0.013 (NS) | 0.471 (**) | 0.369 (*) |
| **R_TN** | 0.229 (NS) | 0.264 (NS) | 0.214 (NS) | 0.215 (NS) | 0.205 (NS) | 0.048 (NS) | -0.08 (NS) |
| **R_NH3_N** | -0.023 (NS) | -0.035 (NS) | -0.072 (NS) | 0.045 (NS) | 0.087 (NS) | -0.245 (NS) | -0.275 (NS) |
| **R_TS** | -0.19 (NS) | -0.155 (NS) | -0.384 (*) | -0.006 (NS) | 0.213 (NS) | -0.316 (*) | -0.286 (NS) |
| **R_SCOD** | -0.198 (NS) | -0.202 (NS) | -0.333 (*) | -0.021 (NS) | 0.13 (NS) | -0.398 (*) | -0.444 (**) |
| **R_pH** | -0.041 (NS) | -0.035 (NS) | 0.082 (NS) | -0.111 (NS) | -0.114 (NS) | 0.029 (NS) | 0.123 (NS) |
| **R_Salinity** | -0.303 (*) | -0.311 (*) | -0.378 (*) | -0.157 (NS) | 0.018 (NS) | -0.474 (**) | -0.377 (*) |
| **R_S_carbohydrate** | -0.35 (*) | -0.354 (*) | -0.318 (*) | -0.288 (NS) | -0.188 (NS) | -0.204 (NS) | -0.115 (NS) |
| **R_S_protein** | -0.239 (NS) | -0.262 (NS) | -0.336 (*) | -0.084 (NS) | 0.048 (NS) | -0.395 (*) | -0.413 (**) |
| **R_T** | -0.049 (NS) | -0.063 (NS) | 0.058 (NS) | -0.133 (NS) | -0.251 (NS) | 0.078 (NS) | -0.047 (NS) |
| **MC** | -0.206 (NS) | -0.193 (NS) | -0.209 (NS) | -0.196 (NS) | -0.107 (NS) | -0.293 (NS) | -0.006 (NS) |
| **GP** | -0.297 (NS) | -0.333 (*) | -0.439 (**) | -0.168 (NS) | -0.014 (NS) | -0.442 (**) | -0.226 (NS) |
| **MP** | -0.347 (*) | -0.378 (*) | -0.472 (**) | -0.226 (NS) | -0.071 (NS) | -0.496 (**) | -0.253 (NS) |
| **AGP** | -0.398 (*) | -0.423 (**) | -0.552 (***) | -0.245 (NS) | -0.07 (NS) | -0.451 (**) | -0.295 (NS) |
| **AMP** | -0.443 (**) | -0.469 (**) | -0.572 (***) | -0.303 (*) | -0.141 (NS) | -0.497 (**) | -0.328 (*) |

*^a^*Spearman's rank correlation coefficients and corresponding significance were reported.

*^b^P* values were adjusted for multiple testing using the FDR method. NS: *P* ≥ 0.05, *: *P* < 0.05, **: *P* < 0.01, ***: *P* < 0.001. Statistically significant correlations (*P* < 0.05) are highlighted in red.

**Reference**

1. Walters W, Hyde Embriette R, Berg-Lyons D et al. Improved bacterial 16S rRNA gene (V4 and V4-5) and fungal internal transcribed spacer marker gene primers for microbial community surveys. mSystems 2015;1(1):e00009-15. 10.1128/msystems.00009-15

2. Du X, Gu S, Zhang Z et al. Spatial distribution patterns across multiple microbial taxonomic groups. Environ Res 2023;223:115470. 10.1016/j.envres.2023.115470

3. Magoc T, Salzberg SL. FLASH: fast length adjustment of short reads to improve genome assemblies. Bioinformatics 2011;27(21):2957-63. 10.1093/bioinformatics/btr507

4. Kong Y. Btrim: A fast, lightweight adapter and quality trimming program for next-generation sequencing technologies. Genomics 2011;98(2):152-3. 10.1016/j.ygeno.2011.05.009

5. Edgar RC. UNOISE2: improved error-correction for Illumina 16S and ITS amplicon sequencing. bioRxiv 2016;081257. 10.1101/081257

6. Rognes T, Flouri T, Nichols B et al. VSEARCH: a versatile open source tool for metagenomics. PeerJ 2016;4:e2584. 10.7717/peerj.2584

7. Wang Q, Garrity GM, Tiedje JM et al. Naive bayesian classifier for rapid assignment of rRNA sequences into the new bacterial taxonomy. Appl Environ Microbiol 2007;73(16):5261-7. 10.1128/AEM.00062-07

8. Katoh K, Misawa K, Kuma Ki et al. MAFFT: a novel method for rapid multiple sequence alignment based on fast Fourier transform. Nucleic Acids Res 2002;30(14):3059-66. 10.1093/nar/gkf436

9. Price MN, Dehal PS, Arkin AP. FastTree 2--approximately maximum-likelihood trees for large alignments. PLoS One 2010;5(3):e9490. 10.1371/journal.pone.0009490

10. Kembel SW, Cowan PD, Helmus MR et al. Picante: R tools for integrating phylogenies and ecology. Bioinformatics 2010;26(11):1463-4. 10.1093/bioinformatics/btq166

11. Faith DP. Conservation evaluation and phylogenetic diversity. Biological Conservation 1992;61(1):1-10. 10.1016/0006-3207(92)91201-3

12. Webb CO, Ackerly DD, McPeek MA et al. Phylogenies and community ecology. Annu Rev Ecol Evol Syst 2002;33:475-505. 10.1146/annurev.ecolsys.33.010802.150448

13. Swenson NG. Functional and phylogenetic ecology in R, 2014 ed. New York, NY: Springer Nature, 2014.

14. Edgar RC. Updating the 97% identity threshold for 16S ribosomal RNA OTUs. Bioinformatics 2018;34(14):2371-5. 10.1093/bioinformatics/bty113
